# Engineered cyclic peptide targeting ITGA5 disrupts tumor–stroma interaction to overcome desmoplasia and resistance in pancreatic ductal adenocarcinoma

**DOI:** 10.1016/j.apsb.2025.10.022

**Published:** 2025-10-24

**Authors:** Deby Fajar Mardhian, Kunal P. Pednekar, Ahmed G. Hemdan, Praneeth Reddy Kuninty, Saadia A. Karim, Sabine de Winter, Josbert M. Metselaar, Jennifer P. Morton, Jai Prakash

**Affiliations:** a Engineered Therapeutics Group, Department of Advanced Organ Bioengineering and Therapeutics, Faculty of Science and Technology, University of Twente, Enschede 7500AE, The Netherlands; b Department of Dental Materials and Technology, Faculty of Dentistry, Padjadjaran University, Bandung 40132, Indonesia; c Department of Pharmacology and Toxicology, Faculty of Pharmacy, Assiut University, Assiut, Egypt; d CRUK Scotland Institute, Glasgow G61 1BD, UK; e ScarTec Therapeutics BV, Horst 2, Enschede 7522LW, The Netherlands; f Institute for Experimental Molecular Imaging (ExMI), RWTH Aachen University Hospital, Aachen 52074, Germany; g School of Cancer Sciences, University of Glasgow, Glasgow G61 1QH, UK; h Department of Medical Biosciences, Radboud University Medical Centre, Geert Grooteplein Zuid 10, Nijmegen 6525 GA, The Netherlands

**Keywords:** Pancreatic stellate cells, Cancer-associated fibroblasts, Pancreatic cancer, Tumor microenvironment, ITGA5, Fibronectin, Peptide, cyAV3.3

## Abstract

The tumor–stroma interaction contributes to the aggressive and resistance nature of pancreatic ductal adenocarcinoma (PDAC), leading to treatment failure. Cancer-associated fibroblasts (CAFs), a key cell type in the stroma, produce abundant extracellular matrix (ECM) and exhibit crosstalk with cancer cells inducing chemoresistance. In this study, we designed a cyclic peptide (cyAV3.3) targeting integrin *α*5 (ITGA5) to disrupt CAF-induced desmoplasia and crosstalk with cancer cells. *In vitro*, cyAV3.3 inhibited the differentiation of pancreatic stellate cells into CAFs and reduced ECM production. In 3D co-cultured human spheroid models, the peptide decreased markers of resistance (ABCG1, BCL2, CXCR4), stemness (WNT1, CD44) and ECM remodeling (COL1A1, MMP2/9, LOX) and enhanced gemcitabine efficacy. *In vivo*, radiolabeled cyAV3.3 exhibited high tumor accumulation and retention following parenteral injections in a co-injection xenograft tumor model. Intriguingly, combination of cyAV3.3 with gemcitabine resulted in improved therapeutic efficacy of gemcitabine in co-injection xenograft and genetically engineered *LSL-Kras*^G12D/+^*LSL-Trp53*^R172H/+^*Pdx1-Cre* (KPC) PDAC models. These effects were attributed to reduced desmoplasia, vasculature compression and enhanced infiltration of cytotoxic T cells and apoptosis. This study presents a novel cyclic peptide inhibiting ITGA5-mediated tumor–stroma interaction and thereby reduce desmoplasia and resistance, ultimately enhancing chemotherapy efficacy in PDAC.

## Graphical abstract

Engineered cyclic peptidomimetic inhibits activation of cancer-associated fibroblasts by targeting ITGA5/FAK axis, leading to reduced desmoplasia. This also inhibits tumor–stroma interaction, reducing stemness and resistance and increasing chemotherapy efficacy.

**Figure:**
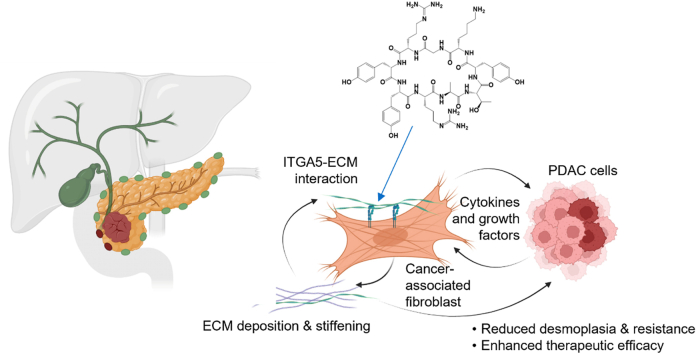

## 1 Introduction

Pancreatic ductal adenocarcinoma (PDAC) is one of the deadliest cancers with a dismal 5-year survival rate of just <11%^1^. Although chemotherapy remains the main treatment for PDAC, it provides only modest survival benefits without significantly improving clinical outcomes. One major reason for treatment failure is the abundant desmoplastic tumor stroma, which promotes tumor progression, confers resistance to cancer therapeutics, and limits drug delivery^2^.

PDAC stroma primarily comprises cancer-associated fibroblasts (CAFs), endothelial cells, immune cells, and extracellular matrix (ECM)^2^. Among these, CAFs are the most dominant cell type, exhibiting diverse phenotypes^3^. CAFs secrete a wide array of growth factors and cytokines, activating both cancer and stromal cells within the tumor microenvironment^4^. They originate from pancreatic stellate cells (PSCs), resident cells of the exocrine pancreas that transform from lipid-storing quiescent cells into myofibroblasts in response to injury or tissue damage^5^. Activated PSCs exhibit increased proliferation and migration and secrete growth factors and cytokines, promoting tumor progression, drug resistance, stemness, and metastasis^5^. Lineage tracing studies have revealed that PSC-derived CAFs contribute to ECM deposition and regulate tissue stiffness and thereby shape the fibrotic microenvironment of PDAC^6^.

CAFs are primarily responsible for the production of interstitial ECM components such as collagens, fibronectin, and tenascin-C in response to transforming growth factor (TGF-*β*). They also drive ECM remodeling by inducing crosslinking and stiffness *via* lysyl oxidase (LOX) enzymes and contraction as well as degradation *via* matrix metalloproteinases (MMPs)^7^^,^^8^. The tumor ECM plays a crucial role in therapeutic resistance by modulating biophysical and biochemical properties^7^. A stiffened ECM promotes tumor-promoting mechanisms such as cancer cell proliferation, metabolic alterations and invasiveness. In addition, the ECM creates a chemo-resistant environment and facilitates hypoxia and hypovascularity^9^. It enhances drug resistance through multiple mechanisms, including supporting cancer stem cell survival and proliferation, inducing stemness *via* integrin receptor signaling, and forming physical barriers that hinder therapeutic delivery.

Cell–ECM interactions are mediated by integrins, a large family of heterodimeric transmembrane receptors composed of *α* and *β* subunits, which have garnered attention as potential therapeutic targets^7^^,^^10^. Integrins bind to ECM proteins and function as bi-directional signaling molecules, regulating cell differentiation, proliferation, migration, and adhesion^11^^,^^12^. Several integrins are overexpressed in various cancers, playing a crucial role in tumor proliferation, migration and therapy resistance *via* ILK/PI3K/AKT and focal adhesion kinase (FAK) signaling^7^^,^^12^^,^^13^. Within the tumor stroma, integrins play a key role in CAF differentiation, ECM production, and cytokine secretion^5^. Moreover, TGF-*β*, a well-known pro-fibrotic growth factor, induces CAF activation, leading to *de novo* ECM synthesis and integrin overexpression. Notably, integrin *α*5*β*1 interacts with fibronectin, creating a self-activating feedforward loop^14^. The role of integrin *α*5 (ITGA5) in PSCs has been shown for their involvement in CAF differentiation, contraction, ECM production and crosstalk with cancer cells in PDAC^15^^,^^16^. In general, *α*5*β*1 has been shown to play crucial role in cancer^17^^,^^18^. Upon binding fibronectin, *α*5*β*1 activates focal adhesion kinase (FAK) and Src kinases triggering PI3/AKT for cell survival, MAPK/ERK for proliferation and RhoGTPases for cytoskeletal remodeling. Also, this receptor has been shown to play an important role in inducing angiogenesis and therapy resistance *via* AKT pathway. Furthermore, there have been developments of antagonists against *α*5*β*1 including antibody (volociximab), peptide (ATN-161) and small molecules (JSM6427, SJ749)^17^. However, to our knowledge, none of them is currently under clinical development due to unreported reasons.

In the present study, we designed a cyclic peptide to effectively block ITGA5 and thereby inhibit tumor-stroma crosstalk in PDAC and improve chemotherapy efficacy. Molecular docking simulations were performed to predict the binding sites and intermolecular interactions and compare their binding and intermolecular energy. We compared the linear and cyclic peptides for the competitive blocking of *α*5*β*1 receptor or PSC with FN. We evaluated the effects of peptides on inhibiting PSC differentiation and ECM deposition and found that the cyclic peptide exhibited superior efficacy compared to its linear counterpart, prompting further studies. We further showed that cyAV3.3 reduced the stroma-induced gene expression linked to therapy resistance, stemness and ECM remodeling in 3D tumor spheroid models, thereby enhancing the cytotoxic effects of gemcitabine. To further understand its behavior *in vivo*, we radiolabeled cyAV3.3 and assessed its pharmacokinetics, tumor uptake and organ distribution *in vivo*. Finally, efficacy studies in tumor models, including co-injection xenografts (tumor cells + PSCs) and genetically engineered KPC (*LSL-Kras*^G12D/+^; *LSL-Trp53*^R172H/+^; *Pdx1-Cre*) mice, revealed that cyAV3.3 enhanced chemotherapy efficacy by reducing desmoplasia, inducing tumor vasculature lumen, and increasing cytotoxic T cells infiltration.

## 2 Materials and methods

### 2.1 Peptides

AV3.3 (Arg-Tyr-Tyr-Arg-Ala-Thr-Tyr), cyAV3.3 (cyclo-(Gly-Arg-Tyr-Tyr-Arg-Ala-Thr-Tyr-Lys)) and scyAV3.3 (scr: Gly-Tyr-Tyr-Thr-Arg-Arg-Ala-Tyr-Lys) were custom synthesized by ChinaPeptide Co., Ltd. (Shanghai, China). The purity of the peptides was assessed by reversed phase high performance liquid chromatography (HPLC, Analytical). The products were stored at −20 °C.

### 2.2 Molecular docking

The molecular docking of AV3.3 and cyAV3.3 with *α*5*β*1 was performed using the Autodock vina version 1.2.0 default protocol, predicting the binding position of the peptides in detail^19^. The rigid structure of the receptor was based on the PDB: 7NWL, which provided us the cryo-electron microscopy structure of active human *α*5*β*1 domain naturally bound to fibronectin at the resolution of 3.1-Å. We removed fibronectin from the structure using PyMOL software ver. 2.5.5 (Schrodinger, LLC), providing us a rigid structure of active *α*5*β*1 receptor. Auto Dock vina analyzes the results of docking simulations, including conformational similarity, visualizing conformations, and interactions between ligands and proteins, as well as the affinity potentials created by Auto Grid, which was placed with the following dimensions: center_*x* = 259.51, center_*y* = 268.06, center_*z* = 258.34 with size_*x* = 37.18, size_*y* = 49.83, size_*z* = 21.90. This grid represents the interaction site of the fibronectin domain 10 to the *α*5*β*1 receptor based on PDB: 7NWL. Docking was performed with exhaustiveness of 8 to achieve the top 5 best poses with the protein. The interaction images were developed using PyMOL software.

### 2.3 Circular dichroism

To characterize the secondary structures of the peptidomimetics, 0.1 mg/mL peptidomimetic in Na_2_HPO_4_ was analyzed using circular dichroism. The assay was conducted using a Hellma Quartz Suprasil (Hellma, Mullheim, Germany) and Jasco J-1500 CD spectrometer (Jasco, Tokyo, Japan). Measurements were performed in the far-UV domain (180–300 nm) at RT with a scanning rate of 100 nm/min and bandwidth of 1 nm. Three spectra per sample were collected and averaged and the signal of the solvent was subtracted to remove background absorption. The observed ellipticity *θ* was converted to the mean residue molar ellipticity [*θ*] using Eq. (1)^20^:

(1) $$\left[\theta \right]=\frac{\theta \times m}{10\times C\times l\times n}$$

*C* is the concentration of peptidomimetic (mg/mL), *m* is the average molecular mass of peptidomimetic, *n* is the number of amino acids in the peptidomimetic, and *l* is the pathway length (cm).

### 2.4 ITGA5 competition study

To examine the affinity of the peptide to the target ITGA5, a 96 well MaxiSorp™ (Thermo Scientific) was coated with 100 μL FN (Advanced Biomatrix) at 1 μg/mL by overnight incubation at 4 °C. Next, the plate was washed with DPBS (Lonza) and blocked with 5% bovine serum albumin (BSA) in DPBS for at least 1 h at RT. In the meantime, mentioned concentrations of the peptides were mixed with 2 μg/mL human integrin *α*5*β*1 in DPBS with Mg^2+^ and Ca^2+^. The mixture was then set to rest for 30 min at RT. After blocking with 5% BSA, the plate was washed with DPBS. The mixture of integrin *α*5*β*1 and peptide was transferred to a well plate and incubated for 1 h at RT. Next, the plate was washed three times with PBS with 0.1% Tween 20 (PBS-T) and then mouse anti-human CD49e (RnD Systems) was added. After 1 h of incubation, plate was washed and the secondary peroxidase labeled antibody was added and incubated for 1 h at RT. After washing with PBS-T, the plate was quickly developed with 3,3′,5,5′-tetramethylbenzidine (TMB) (50 μL per well) and incubated for 5 min at RT in the dark. The reaction was stopped with 3 mol/L H_2_SO_4_ (50 μL per well) and the absorbance was measured at 450 nm with plate reader (Tecan).

### 2.5 Cell adhesion competition study

To analyze the interaction of PSC and FN, a 96 suspension well plate was coated with 100 μL FN (Advanced Biomatrix, Carlsbad, CA, USA) at 1 μg/mL by overnight incubation at 4 °C. Next, the plate was washed with DPBS (Lonza) and blocked with 5% bovine serum albumin (BSA) in DPBS for at least 1 h at RT. In the meantime, a cell suspension of 200,000 cells/mL of TGF-*β* treated PSCs in DPBS with 2 mmol/L Ca^2+^ and 2 mmol/L Mg^2+^ was treated with 50 μmol/L AV3.3 or cyAV3.3 and incubated for 30 min at 37 °C. After washing the plate with DPBS, 100 μL cell suspension was added to each well and then set to rest for 1 h at RT. The plate was then washed with DPBS to remove unattached cells. The remaining attached cells were then fixed with acetone/methanol (1:1) for 30 min at −20 °C. Next, cells were stained with 0.1% (*w*/*v*) crystal violet (Sigma) in 25% methanol at RT. After 20 min, cells were washed with water and lysed with 0.5% (*w*/*v*) SDS. The degree of cell attachment was measured by the absorbance of the crystal violet at 600 nm using Tecan Infinite M200 plate reader (Tecan, Mannendorf, Switzerland). In other plate, cells were washed with DPBS after fixation and then stained with DAPI. Images were developed using EVOS fluorescence microscope (Thermo Scientific).

### 2.6 Cells

Primary human pancreatic stellate cells (hPSCs, ScienCell, Carlsbad, CA, USA) and pancreatic cancer cells PANC-1 and MIA PaCa-2 (ATCC, Manassas, VA, USA) were cultured as described previously^21^.

### 2.7 Tumor spheroid formation

Homo-spheroids containing hPSCs, PANC-1, or MIA PaCa-2, and hetero-spheroids containing 1:1 combination of hPSCs and PANC-1/MIA PaCa-2 were generated in a 96-well round bottom plates precoated with 1% Pluronic F-127 (Sigma–Aldrich). The self-assembled spheroids were exposed to cyAV3.3, gemcitabine, or both after 3 days and 6 days. Before each treatment, images of spheroids were made using inverted microscope. The diameters of spheroids were measured digitally using ImageJ software. After 9 days, spheroids were moved from the wells to a fresh 96-well plate. CellTiter-Glo 3D reagent (Promega, WI, USA) was added to each well and the luminescence signal was measured after 30 min using Varioskan LUX bioluminescent reader (Thermo Scientific) to assess the viability.

### 2.8 Animal experiments

The experimental protocols were approved by the local animal welfare and ethical board of the institutes. The ethical approval by the Dutch Central Animal Welfare (AVD1100020174305). Animals were housed in individually ventilated cages and fed ad libitum. The housing temperature was controlled at 20–24 °C and humidity of 40%–60% with a 12 h light and 12 h dark cycle was maintained.

### 2.9 Human co-injection xenograft tumor model

Six-week-old male CB17 SCID mice (Janvier Labs, Le Genest-Saint-Isle, France) were subcutaneously injected with a 2:1 mixture of 4 × 10^6^ human PSCs and 2 × 10^6^ PANC-1 or MIA PaCa-2 cells. Tumor growth was assessed twice a week. Tumor-bearing mice (±100 mm^3^) were treated with phosphate buffered saline (PBS), cyAV3.3, gemcitabine, or combination of gemcitabine and cyAV3.3 intraperitoneally (i.p). Animals were euthanized, tumors and other organs were harvested and embedded in Cryomatrix (Thermo Scientific) before being snap frozen in cold 2-methyl butane (Thermo Scientific) and stored at −80 °C until analyses.

### 2.10 cyAV3.3 biodistribution study in co-injection xenograft PDAC model *in vivo*

Radiolabeling of cyAV3.3 and detailed animal experiments are described in Supporting Information In Tumor-bearing mice, ^125^I-cyAV3.3 was injected i.p or intravenously (5 μg/kg) with/without cold cyAV3.3 (i.v. 5 mg/kg) 10 min before ^125^I-cyAV3.3. 30–60 μL of blood was withdrawn *via* retro-orbital plexus and the radioactivity was measured in a Gamma counter (Wallace Wizard 2470, PerkinElmer).

### 2.11 cyAV3.3 radiolabeling and binding assay

In a 1.5 mL low-binding microtube, 69.4 nmol Iodogen (Thermo Scientific) was coated in 100 μL of dichloromethane and kept at −20 °C until use. In the 1.5 mL low-binding microtube coated with Iodogen, 25 μg of cyAV3.3 (21.6 nmol) was added to 70 μL of 0.1 mol/L phosphate buffer pH 7.4 and 3.5 mCi (129.5 MBq) of Na^125^I (PerkinElmer) solution. The reaction was allowed to stir for 20 min at RT. Thereafter, 90 μL of PBS 1 × /0.1% Tween was added in order to obtain a final volume of 200 μL. The labeling medium was stirred for 5 min and was purified by RP-HPLC using a C18 column (YMC-Triart, 5 μm, 4.6 mm × 250 mm), applying a linear gradient of 0%–90% of acetonitrile/water containing phosphoric acid (7.6 mmol/L) for 50 min (flow rate of 1 mL/min; Gamma radioactivity detection and UV detection at 220 nm).

The affinity of radiolabeled cyAV3.3 on human primary pancreatic stellate cells (hPSCs) was investigated by carrying out a saturation assay in order to determine the equilibrium dissociation constant (*K*_d_). hPSCs (1 × 10^6^ cells in 50 μL sample buffer) were incubated with increasing concentrations of radiolabeled peptidomimetic solution in a final volume of 150 μL for 90 min at RT with agitation. The non-specific binding was determined by co-incubating an excess of unlabeled peptidomimetic (200-fold molar excess). Following incubation, reaction mixtures were overlaid onto 200 μL of a dibutylphtalate oil cushion and centrifuged in microfuge tubes at 10,000×*g* (Thermo Scientific) for 3 min. Tubes were frozen in liquid nitrogen and the tips of the tubes containing the cell pellets were cut off for determination of radioactivity using a Gamma counter. Non-specific binding was determined by measuring the bound radioactivity of five-point concentrations of radiolabeled cyAV3.3 containing 200-fold excess of unlabeled peptidomimetic.

### 2.12 cyAV3.3 biodistribution study in co-injection xenograft tumor model *in vivo*

The experimental protocol was approved by the local ethical committee of Chelatec SAS (Saint-Herblain, France). Six-week-old male CB17 SCID (CB17/Icr-PrkdcSCID/lcrIcoCrl mice (Charles River Labs, Saint-Germain-Nuelles, France) were subcutaneously injected with a 2:1 mixture of 4 × 10^6^ hPSCs and 2 × 10^6^ PANC-1 cells. Tumor growth was assessed twice a week or by measuring the size using Vernier caliper and tumor volumes were calculated according to Eq. (1):

Volume = 0.5236 × Length × Width × Height (1)

The study was carried out in three separate experiments where at study n°1 mice received ^125^I-cyAV3.3 by IP route at 5 μg/kg dose, at study n°2 mice received ^125^I-cyAV3.3 by IV route at 5 μg/kg dose, at study n°3 mice received cold cyAV3.3 by IV route at 5 mg/kg dose and followed by IV injection of ^125^I-cyAV3.3 by IV route at 5 μg/kg dose after 10 min. At the intermediate time point, approximately 30–60 μL of blood was sampled from the retro-orbital plexus on anesthetized mice (with isoflurane gas). At the terminal time point, the blood samples were obtained from exsanguination *via* intracardiac puncture on anesthetized mice by intraperitoneal injection of a mixture of ketamine/xylazine. Blood samples were collected into preweighed Microvette tubes with clotting activator (Sarstedt) and the radioactivity was measured in a Gamma counter. Then, blood samples were processed for serum (centrifugation for 5 min at 10,000×*g*) and serum samples were also analyzed for their radioactivity in a Gamma counter. At the terminal time point, the animals were anaesthetized, the organs were harvested, rinsed with NaCl 0.9%, blotted dry and weighed. The counting of tissue radioactivity was performed in an automatic gamma counter (Wallace Wizard 2470–Perkin Elmer) calibrated for Iodine-125 radionuclide.

### 2.13 Genetically engineered KPC mouse model

*LSL-Kras*^G12D/+^; *LSL-Trp53*^R172H/+^; *Pdx1-Cre* (KPC) mice were bred and maintained, on a mixed background, at the CRUK Scotland Institute. Mice were palpated until an abdominal tumor was detected (between 12 and 28 weeks of age) and were then imaged under anesthesia by 3D ultrasound imaging using the VisualSonics Vevo 3100 system with the MX550D transducer (FujiFilm). Following confirmation of PDAC of 3–6 mm, mice of both sexes were enrolled on treatment with cyAV3.3 25 mg/kg i.p. b.i.w. and gemcitabine (100 mg/kg i.p. b.i.w., *n* = 6). No mice were excluded from the study. Mice treated with gemcitabine alone were used as control group (*n* = 6). Follow-up imaging to monitor tumor volume was performed weekly until endpoint was reached (moderate symptoms: abdominal distension, loss of body conditioning, jaundice, hunching, or reduced movement). Upon post-mortem, gross pathology was examined and PDAC and organs harvested and fixed in 10% neutral buffered formalin prior to paraffin embedding and sectioning for histological and IHC analysis. Kaplan–Meier and Log-Rank analysis was used for statistical assessment of survival. Analysis of ultrasound images was performed by researchers blinded to treatment arm using VisualSonics VevoLab software (version 3.1.1). In 3D mode, stacked images of the tumor were imported, and tumor border annotated, allowing a 3D volume analysis.

### 2.14 Graphs and statistical analyses

All statistical analyses were performed using GraphPad Prism version 10 (GraphPad Software Inc., San Diego, CA, USA), which are described in the figure captions. The minimal significance is considered at *P* < 0.05 and *P* values are mentioned in the figure legends.

## 3 Results

### 3.1 Targeting ITGA5 using cyclic peptide

**Figure 1:**
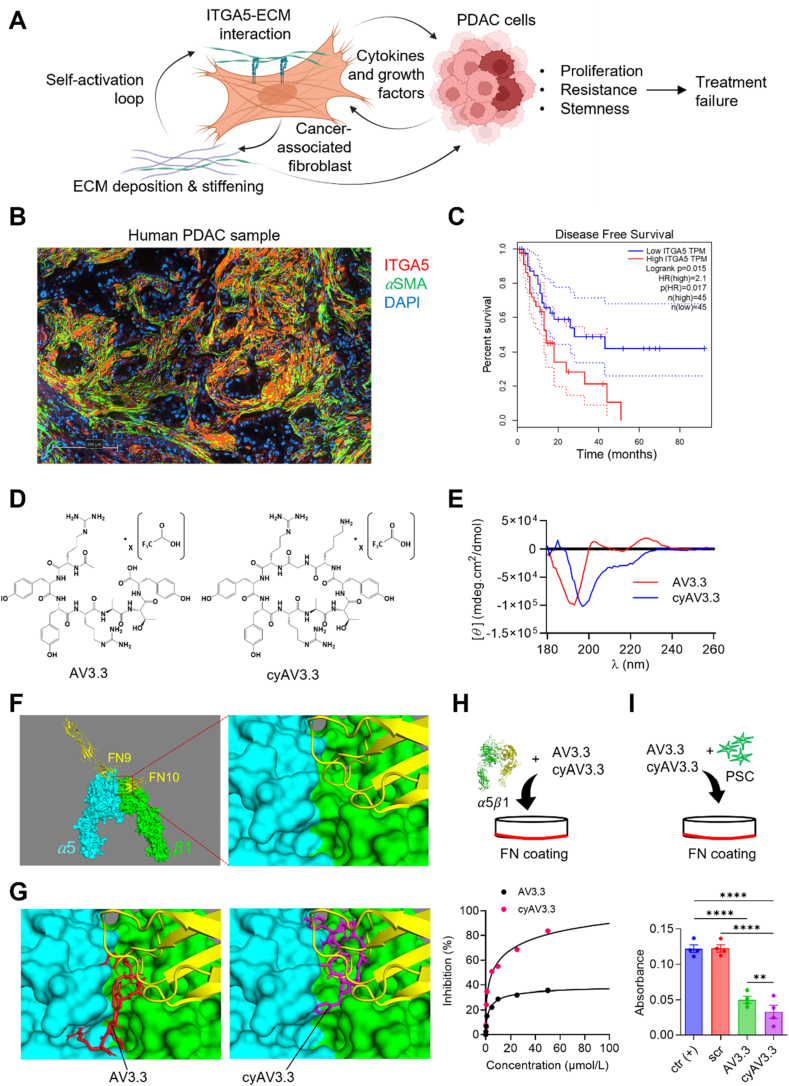
Targeting ITGA5 using peptides. (A) Schematic diagram showing the ITGA5–ECM interaction in the activation of CAFs and crosstalk with cancer cells. Created with Biorender.com. (B) Immunofluorescence double staining for ITGA5 and *α*-SMA in human PDAC sample. (C) Disease-free survival curve showing that the overexpression of ITGA5 is associated with poor disease-free survival. (D) Chemical structures of linear peptide AV3.3 and cyclic peptide cyAV3.3. (E) Circular dichroism spectrum of the linear and cyclic peptide. (F) Schematic representation of ITGA5 blocking by the peptides to prevent interaction with fibronectin (FN). (G) Docking images showing binding of AV3.3 and = cyAV3.3 with ITGA5 obtained using Autodock software. (H) Fit curves line graphs showing the competition binding inhibition (%) of human recombinant *α*5*β*1 incubated with different concentrations (0.1, 0.5, 1, 5, 10, 20, and 50 μmol/L) of AV3.3 or cyAV3.3 to compete for binding to the FN coated on the plate. (I) The bar graph shows the absorbance value of attached cells stained with crystal violet on the FN-coated plate with/without 50 μmol/L peptides. Data represent means ± SEM, *n* = 3. Statistical comparisons were performed using one-way ANOVA corrected for multiple comparisons with Holm-Sidak test. ∗∗∗∗*P* < 0.0001.

CAFs produce the ECM such as fibronectin *de novo*, which *via* interaction with ITGA5 receptor, creates a self-activation loop, re-stimulating themselves. Activated CAFs secrete cytokines and growth factors as well as stiffen ECM, which stimulate cancer cells inducing their proliferation, resistance and stemness (Fig. 1A). Immunofluorescence staining shows that ITGA5 is overexpressed on *α*-SMA + CAFs in human PDAC tissue (Fig. 1B). Also, higher expression of ITGA5 is associated with the poor disease-free survival in PDAC patients, as shown with Kaplan–Meier graph created using GEPIA2 (Gene Expression Profiling Interactive Analysis 2)^22^ (Fig. 1C). Based on the protein–protein interaction studies between human FN and *α*5*β*1 receptor^23^, using *in silico* tools, we designed a linear peptide AV3.3 and a cyclic peptide cyAV3.3 (Fig. 1D). The binding sequence RYYRITY from human FN-III domain 10 were modified using alanine replacement assay, which resulted into RYYRATY (AV3.3) peptide sequence (Supporting Information Fig. S1A).

We engineered the cyclic peptide through head-to-tail cyclization by introducing glycine at the N-terminal to limit steric hindrance and lysine at the C-terminal for cyclization. To evaluate the changes in structures, circular dichroism spectrometry was performed, which showed a shift in structure from random organization (linear AV3.3) to antiparallel *β* strand (cyAV3.3) (Fig. 1E). A previous study has studied the crystal structure of FN with *α*5*β*1, which showed FN binding induces integrin opening rather than FN binding occurring as a consequence of integrin opening^24^. Fig. 1F shows the interaction of FN with *α*5*β*1 based on x-ray crystallography data. *In silico*, we performed docking studies of these peptides with active *α*5*β*1 (Protein Data Bank (PDB): 7NWL, without FN) using Autodock vina software to predict the binding pocket (Fig. 1G). Fig. S1B shows the placement of grid to perform the docking analysis, which was based on the binding site of FN domain 10 to *α*5*β*1, as determined from PDB: 7NWL. The cluster analysis of top conformations revealed that both AV3.3 and cyAV3.3 peptides bound to the binding site of *α*5*β*1 at the interjunction of *α*5 and *β*1 subunits, which forms the pocket of the FN binding site (Fig. 1G and Fig. S1C).

Furthermore, cyAV3.3, in comparison to AV3.3, showed a better fit into the FN binding pocket of the *α*5*β*1 receptor, as shown in Fig. 1G. The interaction data revealed a number of amino acids, both from *α*5 and *β*1 chains having interactions with AV3.3 and cyAV3.3. Data indicate that both peptides bind to some common amino acids such as Asp227 from the *α*5 chain and Ser227 from the *β*1 chain, but also to other amino acids (Fig. S1D, Supporting Information Table S1). While the docking tools provide a prediction for interactions, docking of cyclic peptides using these tools is not perfect which may lead to some uncertainty and need further investigation.

We also examined the stability of the peptides in the human plasma and found that there was a significant improvement in the stability of the cyclic peptide (Supporting Information Fig. S2). To examine whether peptides block ITGA5 interaction with its natural ligand fibronectin (FN), human recombinant *α*5*β*1 was co-incubated with increasing concentrations of the peptides in FN-coated plates.

Interestingly, both peptides blocked the FN-*α*5*β*1 interaction with their increasing concentrations with inhibition constant (*K*_i_ value, AV3.3: 2.89 μmol/L and cyAV3.3: 16.9 μmol/L) (Fig. 1H). Interestingly, cyAV3.3 blocked the interaction significantly stronger than AV3.3, indicating its superiority over the linear peptide, which is most likely caused by its macrocyclic effect thereby physically blocking the FN–ITGA5 interaction^25^. Also, cyAV3.3 blocked the binding of PSC to FN significantly higher than AV3.3 at 50 μmol/L concentration (Fig. 1I). Of note, scrambled cyclic peptide (scr) did not show any blocking.

### 3.2 Inhibitory effect of cyAV3.3 on differentiation of pancreatic stellate cells

**Figure 2:**
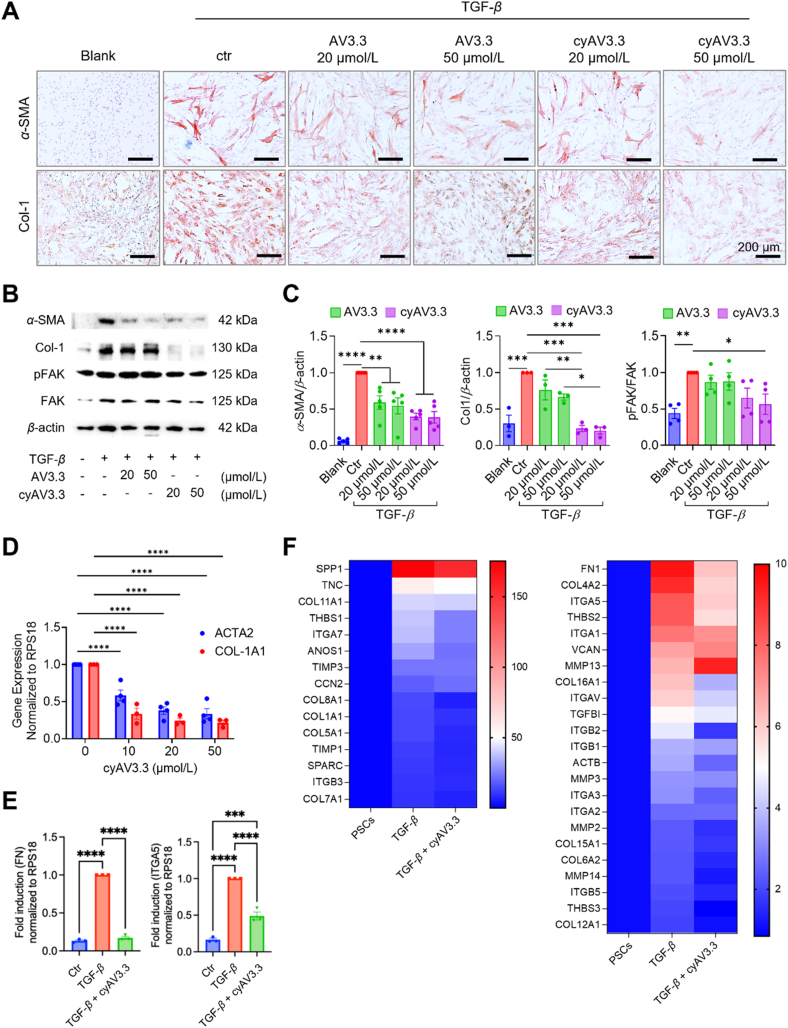
*In vitro* comparative effects of cyclic (cyAV3.3) *versus* linear peptide (AV3.3). (A) Representative microscopic images of immunocytochemical staining showing the effect of peptides at 20 and 50 μmol/L on the expression of *α*-SMA and collagen type 1 in hPSC activated with 5 ng/mL TGF-*β* for 48 h compared to untreated hPSC. Scale bar: 200 μm. (B) Western blot and (C) quantification showing the effect of peptides at 20 and 50 μmol/L on the levels of pFAK, *α*-SMA, and Col-1a1 in hPSC activated with 5 ng/mL TGF-*β* for 48 h compared to untreated hPSC. Statistical comparison was performed using one-way ANOVA corrected for multiple comparisons with the Holm-Sidak test. (D) Effect of cyAV3.3 on the TGF-*β*-induced gene expression of ACTA2 and COL-1A1 in hPSCs dose dependently. Heatmap showing differential gene expression of adhesion molecules and ECM using human ECM RT^2^ Profiler PCR array in hPSC activated by TGF-*β*. Treatment with cyAV3.3 (50 μmol/L) downregulated hPSC activation by TGF-*β*. (E) Gene expression analysis using quantitative polymerase chain reaction (qPCR) for ITGA5, and FN in hPSCs activated by TGF-*β*. Treatment with cyAV3.3 (50 μmol/L) downregulated hPSC activation by TGF-*β*. (F) Heatmap shows the expression levels of 38 genes in PSCs treated with TGF-*β* and their downregulation after treatment with cyAV3.3. Data represent means ± SEM from at least three independent experiments. Statistical comparison was performed using one-way ANOVA corrected for multiple comparisons with the Holm-Sidak test. ∗*P* < 0.05, ∗∗*P* < 0.01, ∗∗∗*P* < 0.001, ∗∗∗∗*P* < 0.0001.

We investigated the inhibitory effect of both cyclic and linear peptides on PSC activation and signaling. To achieve this, PSCs were seeded and treated with TGF-*β* and peptides for 48 h (protein expression) or 24 h (gene expression). We also tested the effect of the peptide on the cell viability using Alamar blue assay and found no toxicity (Supporting Information Fig. S3A). Interestingly, both AV3.3 and cyAV3.3 inhibited the activation of PSCs by human recombinant TGF-*β*1, as shown by the reduced expression of *α*-SMA and collagen-1 stainings in a dose-dependent manner (Fig. 2A). To quantitate that, we performed Western blot analysis and confirmed the observed effect *via* immunostaining (Fig. 2B and C; Fig. S3B). The details of the antibodies used can be found in Supporting Information Table S2. Interestingly, the inhibitory effect of cyAV3.3 was much stronger than AV3.3, indicating the superior potency of the cyclic peptide compared to the linear peptide. Besides the activation markers, we also examined the effect on downstream signaling *p*-FAK and found more inhibition by cyclic version. After obtaining these effects, we focused on cyAV3.3 and examined its inhibitory effects on TGF-*β*1-stimulated PSCs using qPCR and an RT^2^ profiler PCR array for ECM and adhesion receptors (Fig. 2D). We found that cyAV3.3 inhibited both ACTA2 and COL-1A1 concentration dependently (Fig. 2D). Also, cyAV3.3 significantly inhibited the gene expression of FN and ITGA5 (Fig. 2E). Furthermore, the heatmap shows the expression levels of 38 genes including *SPP1*, *THBS1* and *CCN2* (involved in cell–cell and cell–ECM crosstalk), ECM proteins (TNC, SPARC, collagens, FN1, VCAN), integrins (ITGA3, ITGA5, ITGA7, ITGAV, ITGB1, ITGB3, ITGB5) and MMPs were upregulated after treatment with TGF-*β* in PSCs. The details of primers for all genes can be found in Supporting Information Table S3. Interestingly, treatment with cyAV3.3 downregulated all the upregulated genes, as evident from the change in heatmap color (Fig. 2F). Many genes related to ECM and adhesion proteins which were marginally enhanced by TGF-*β*, were reduced by cyAV3.3. In contrast, cyAV3.3 induced the gene expression of ECM-degrading proteins such as MMP10 and ADAMTS-13 (Supporting Information Fig. S4). Overall, these data reveal that blockade of ITGA5 with cyAV3.3 inhibits TGF-*β*-induced differentiation and the myofibroblastic phenotype of PSCs.

### 3.3 Effect of cyAV3.3 on the mRNA expression in 3D stroma-rich spheroid model

**Figure 3:**
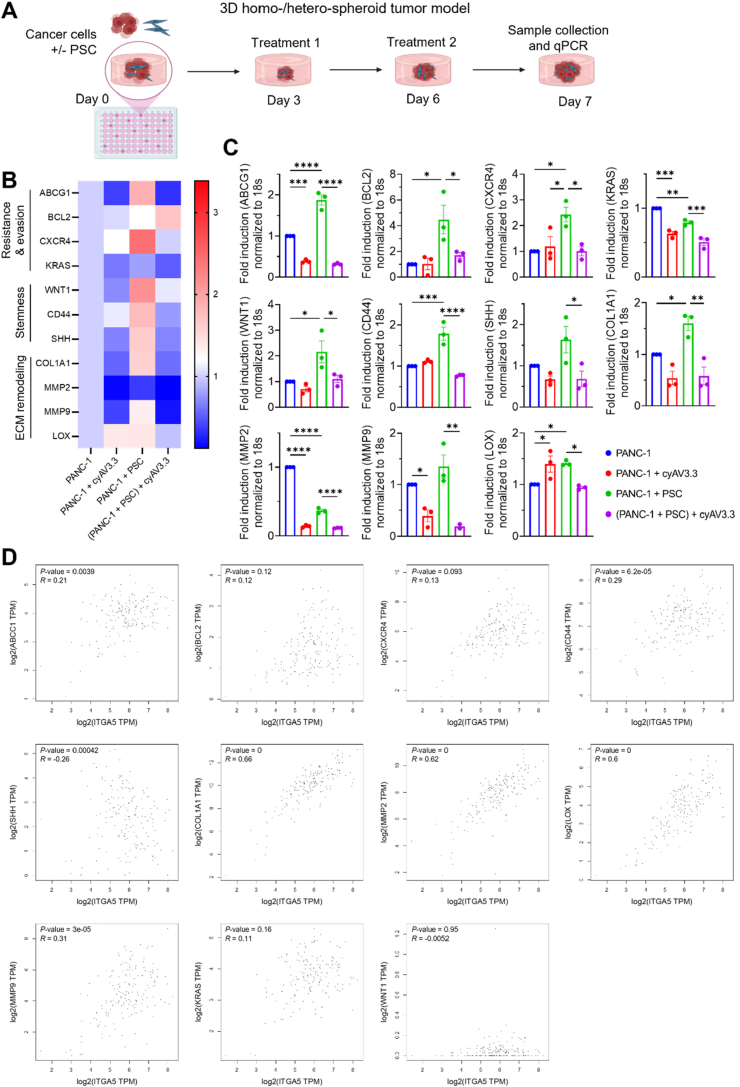
Effect of cyAV3.3 on the gene expression in 3D heterospheroids. (A) Schematic representation of formation of homo- or hetero-spheroids comprised of PANC-1 PSCs^−/+^ in a round bottom 96-well plate. Created with Biorender.com. Heatmap (B) and bar graphs (C) showing the changes in the gene expression levels in PANC-1 homospheroids and PANC-1 + PSC heterospheroids after the treatment with cyAV3.3. Data represent mean ± SEM for *n* = 3 individual experiments. Statistical analysis was performed using ordinary one-way ANOVA corrected for multiple comparisons with Holm-Sidak test. ∗*P* < 0.05, ∗∗*P* < 0.01, ∗∗∗*P* < 0.001, ∗∗∗∗*P* < 0.0001. (D) Pearson correlation of ITGA5 with various gene markers in PDAC from publicly available data using GEPIA2 online tool.

Cancer cells establish a crosstalk with PSCs in juxtracrine and autocrine fashion, resulting in the activation of PSCs into CAFs as well as induction of proliferation, resistance, and stemness in cancer cells^26^. To examine the effect of cyAV3.3 on the tumor–stroma interaction, we developed 3D tumor spheroids of human pancreatic tumor cell line PANC-1 alone (homospheroids) or of co-culturing PANC-1 with human PSCs (heterospheroids; Fig. 3A). We found that on Day 3 spheroids were formed and after that we treated them with the peptide on Days 3 and 6 and examined the gene expression on Day 7 using qPCR for resistance and evasion, stemness and the ECM remodeling. Interestingly, heterospheroids showed an enhanced expression of genes related to drug resistance (*ABCG1*, *BCL2*, *CXCR4*), stemness (*WNT1*, *CD44*, *SHH*) and ECM remodeling (*COL-1A1*, *MMP9*, *LOX*) (Fig. 3B and C). These data are in line of the previous studies showing ITGA5+ myofibroblasts can induce stemness in cancer cells in hepatocellular carcinoma^27^.

Surprisingly, MMP2 expression level reduced in heterospheroids compared to homospheroids, which can be explained by low expression of MMP2 in PSCs and thereby overall decreased collective expression level in heterospheroids. Intriguingly, the gene expression levels related to resistance, stemness and ECM were significantly reduced by cyAV3.3 treatment in heterospheroids (Fig. 3B and C). These data are in line of a recent study in which they showed that blockade of *α*5*β*1 and *α*v*β*3 using bispecific antibody reduces CAF-induced stemness in PDAC cells^27^. In addition, some genes including ABCG1, KRAS, MMP2, MMP9 were inhibited by cyAV3.3 in homospheroids, which indicates the direct effect of ITGA5 inhibition on resistance in tumor cells (Fig. 3B and C). As shown in Fig. 3D, we furthermore correlated these genes with ITGA5 in publicly available human PDAC data using GEPIA2^22^. Some genes related to resistance and stemness (ABCG1, CD44) and ECM (COL1A1, MMP2, 9, LOX) showed positive correlation with ITGA5. These data along with the effect of cyAV3.3 on the gene expression in 3D spheroids indicate that ITGA5 regulates mechanisms associated with resistance, stemness and stroma.

### 3.4 Effect of cyAV3.3 on the effect of gemcitabine in 3D spheroid model

**Figure 4:**
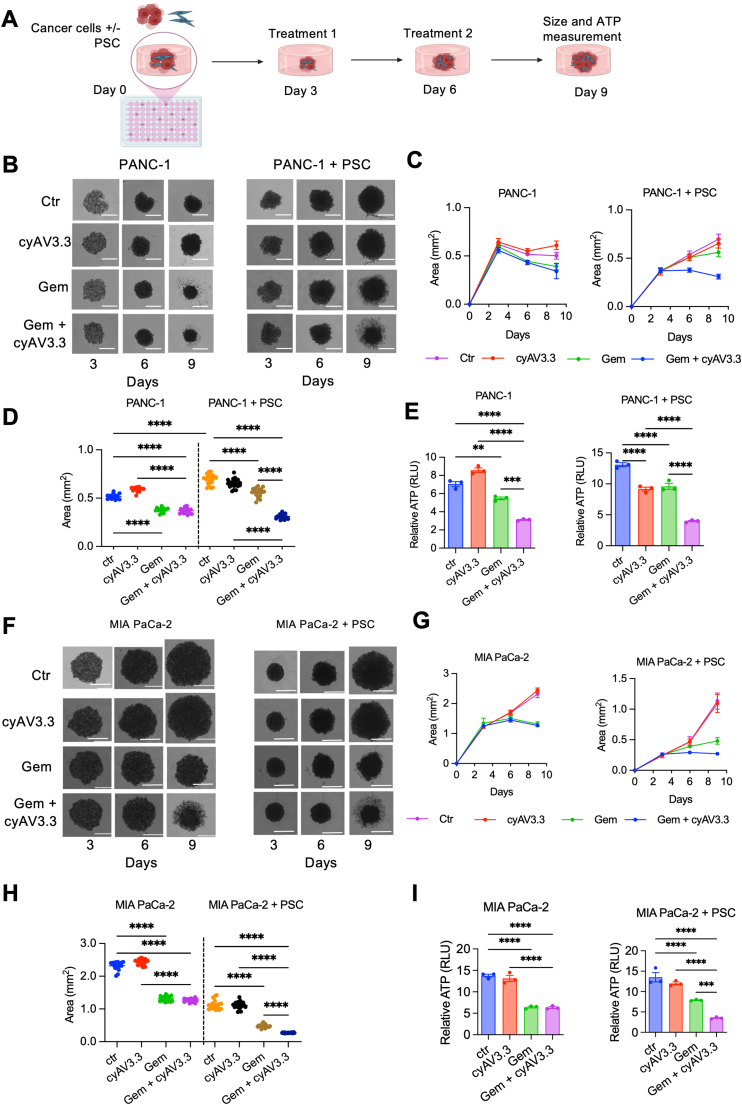
Effect of cyAV3.3 on the tumor stroma in 3D heterospheroids. (A) Schematic representation of treatment of cyAV3.3 and/or gemcitabine in homo- and hetero-spheroids. Created with Biorender.com. (B) Representative images showing growth of PANC-1 spheroids with or without PSCs on Days 3, 6 and 9. Scale bars are 1 mm and all images were captured at the same magnification. Spheroid growth curves (C), the spheroid size on Day 9 (D) and relative ATP levels (E) of homo- and hetero-spheroids after the treatment with cyAV3.3 and/or gemcitabine. (F) Representative images showing growth of PANC-1 spheroids with or without PSCs on Days 3, 6 and 9. Scale bars are 1 mm and all images were captured at the same magnification. Spheroid growth curves (G), the spheroid size on Day 9 (H) and relative ATP levels (I) of homo- and hetero-spheroids after the treatment with cyAV3.3 and/or gemcitabine. Data represent means ± SEM and the statistical analysis was performed using ordinary one-way ANOVA corrected for multiple comparisons with Holm-Sidak test. ∗∗∗*P* < 0.001, ∗∗∗∗*P* < 0.0001. *n* = 20 in Fig. 4C, D, G, H *n* = 3 in Fig. 4E–I with each replicate containing 3 spheroids pooled from prior experiments.

To understand the effect of cyAV3.3 on the effect of chemotherapy, we treated the spheroids on Days 3 and 6 and measured the size on these days, followed by measurement of size and ATP activity on Day 9 (Fig. 4A). As shown in Fig. 4B and C, PANC-1 homospheroids did not grow from Days 3 to day 9, while PANC-1 + PSC heterospheroids grew rapidly.

Treatment with gemcitabine reduced the growth of both homo- and hetero-spheroids. Importantly, the combination of gemcitabine and cyAV3.3 showed synergistic effects only in heterospheroids (Fig. 4B–D). Data from Day 9 demonstrated that the combination of gemcitabine and cyAV3.3 reduced the size of heterospheroids as well as the ATP activity (Fig. 4E). However, these effects were not observed in homospheroids, confirming the stroma-specific effects of cyAV3.3. Compared to PANC-1 spheroids, MIA PaCa-2 spheroids were much larger in size and grew consistently while co-cultured spheroids of MIA PaCa-2 were more compact on Day 3 and then grew until Day 9 (Fig. 4F). These data demonstrate the cell–cell interaction differences between tumor cells. Similar to the effects in PANC-1 model, combination of cyAV3.3 with gemcitabine improved the cytotoxic effects (Fig. 4G–I). Altogether, these data show that the enhanced effect of gemcitabine in combination with cyAV3.3 is mainly observed in stroma-rich hetero-spheroids, indicating the anti-stromal role of cyAV3.3.

### 3.5 Pharmacokinetics and biodistribution of cyAV3.3 in tumor xenograft model

**Figure 5:**
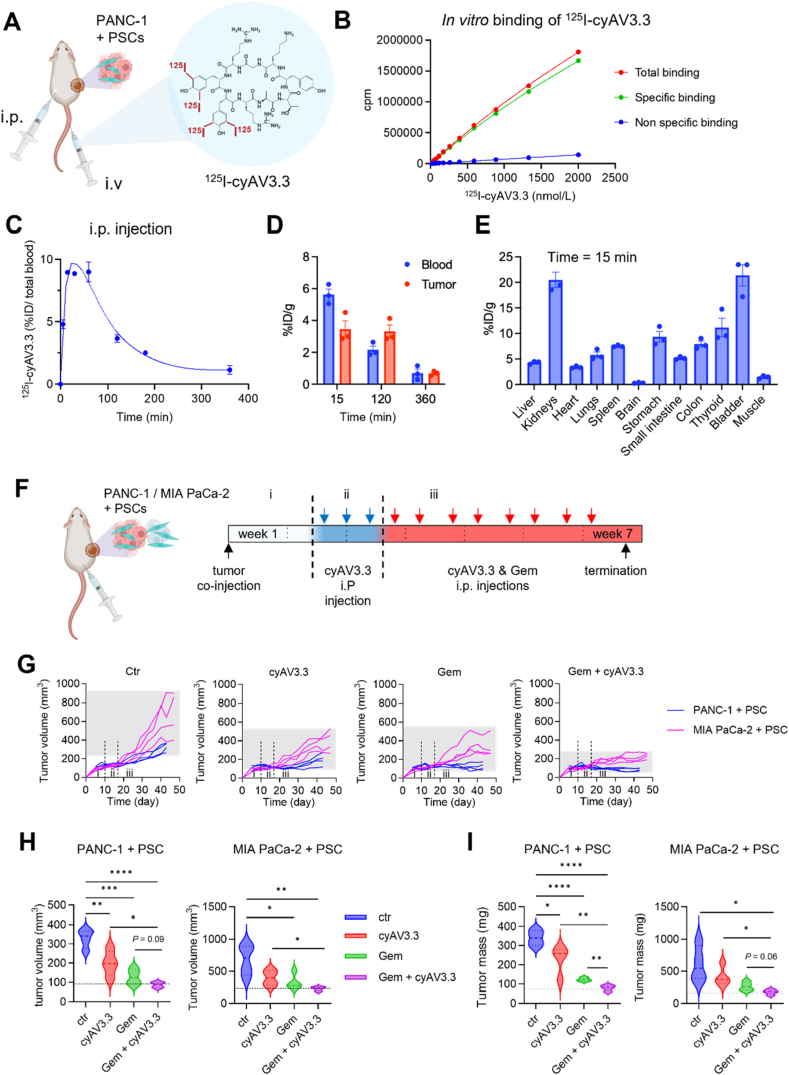
Pharmacokinetics (PK), biodistribution and efficacy of cyAV3.3 in co-injection tumor xenograft model(s). (A) Schematic representation showing radiolabeled cyAV3.3 (^125^I-cyAV3.3) injected into PANC-1 + PSCs co-injection xenograft tumor model in CB17 SCID mice. Image is created using Biorender.com. (B) Binding affinity of ^125^I-cyAV3.3 on PSCs *in vitro*. Non-specific binding refers to the binding after blockade with an excess of cold cyAV3.3. (C) PK results of ^125^I-cyAV3.3 in blood upon i.p. injection (D) %ID/g of ^125^I-cyAV3.3 recovered in blood and tumor after 15, 120 and 360 min. (E) %ID/g of ^125^I-cyAV3.3 recovered in various tissues/organs after 15 min of the i.p. injection. Data represent means ± SEM for *n* = 3. (F) Regimen of treatment in co-injection pancreatic tumor model in CB17 SCID mice. (G) Individual tumor growth curves for different treatment groups. (H) Tumor volume of isolated tumors in PANC-1 + PSC (left) and MIA PaCa-2 + PSC (right) tumor bearing mice. (I) Weight of isolated tumors in PANC-1 + PSC (left) and MIA PaCa-2 + PSC (right) tumor bearing mice. Data represent the box-and-violin plot for *n* = 4 per group for each tumor type. Statistical analysis was performed as ordinary one-way ANOVA for multiple comparisons corrected with Holm-Sidak test. ∗*P* < 0.05, ∗∗*P* < 0.01, ∗∗∗*P* < 0.001, ∗∗∗∗*P* < 0.0001.

To study the pharmacokinetics of cyAV3.3, we radiolabeled cyAV3.3 with iodogen method taking advantage of the tyrosine amino acids in the sequence (Fig. 5A). The labeling resulted in a yield of 64% and >99% purity with specific activity of 2200 Ci/mmol after HPLC purification (Supporting Information Fig. S5A).

No degradation of the radiolabeled peptide (^125^I-cyAV3.3) was observed after 3 days of storage at −20 °C (99% after 9 days) (Fig. S5B). The binding experiment of ^125^I-cyAV3.3 was performed in human PSCs. Data showed that there was a concentration-dependent increase in the binding and addition of 200-fold excess unlabeled cyAV3.3 resulted in a strong blocking of the binding (Fig. 5B), resulting in an estimated *K*_d_ value of 9.28 μmol/L. Then, we performed a PK study in a co-injection (PANC-1 + PSCs) mouse xenograft model after i.v. and i.p. injection (Fig. 5A). We examined the PK and biodistribution after i.p. injection, which is the route for our treatments *in vivo* in tumor models. We found that the peptide reached the *C*_max_ within 15 min after the i.p. injection and then followed the elimination route similar to the i.v. profile (Fig. 5C, Supporting Information Fig. S6A). We followed the distribution in blood and tumors at different timepoints and found nearly 4% ID/g accumulation in tumors after 15 min which stayed the same for at least 2 h and dropped to 1.1% ID/g after 6 h (Fig. 5D). The early biodistribution data at *t* = 15 min showed that there was a rapid elimination *via* kidneys, indicated by high values in kidneys and bladder (Fig. 5E). After i.v. injection, we found that the peptide was rapidly eliminated from the circulation and accumulated into the tumor (about 4% ID/g) within 3 h of the injection (Fig. S6A and S6B). In the organ distribution results kidneys and bladder showed high levels indicating the elimination *via* kidneys (Fig. S6C). The thyroid also showed high values likely due to the accumulation of released iodine. In addition, when we pre-treated the mice with excess of cold cyAV3.3 10 min before the radiolabeled peptide, it prolonged the plasma residence and increased the tumor accumulation (Fig. S6D and S6E), which is likely due to the blockade of unspecific binding sites at the injection site and elsewhere in the body. These data show that cyAV3.3 rapidly accumulates in the tumor after i.p. injection, which warrants efficacy studies in tumor models.

### 3.6 Effect of cyAV3.3 on efficacy of gemcitabine in co-injection xenograft models

After confirming the effect in 2D and 3D models and better understanding the PK and tumor accumulation, we studied the effect of cyAV3.3 on the efficacy of gemcitabine *in vivo*. To mimic tumor heterogeneity, we developed a mixed tumor model in which different tumor human PDAC cell lines (PANC-1 or MIA PaCa-2) were co-injected with human PSCs in SCID mice (Fig. 5F). We found that tumor in the MIA PaCa-2+ PSCs co-injection model showed a more rapid growth compared to the PANC-1+PSC model (Fig. 5G). We used the tumor priming approach, which aims to reprogram the tumor microenvironment before treatment with chemotherapy. After tumors reached 100 mm^3^ size, mice were treated with cyAV3.3, followed by combination therapy with gemcitabine, as detailed in the Materials and Methods section. We found that treatment with cyAV3.3 alone reduced the tumor size by nearly 30%, probably due to its inhibitory effect on the tumor stroma (Fig. 5G and H). Such an effect was also observed in our co-treatment in 3D heterospheroid *in vitro* model (Fig. 4). The combination treatment reduced the tumor growth by nearly 75% compared to monotreatment of gemcitabine (60%) (Fig. 5H). These effects were also seen in the isolated tumors mass (Fig. 5I). Of note, cyAV3.3 did not show any obvious signs of toxicity in terms of a possible negative effect on body mass or organ mass (Supporting Information Fig. S7).

### 3.7 Effect of cyAV3.3 on collagen deposition and tumor vessels in co-injection xenograft model

**Figure 6:**
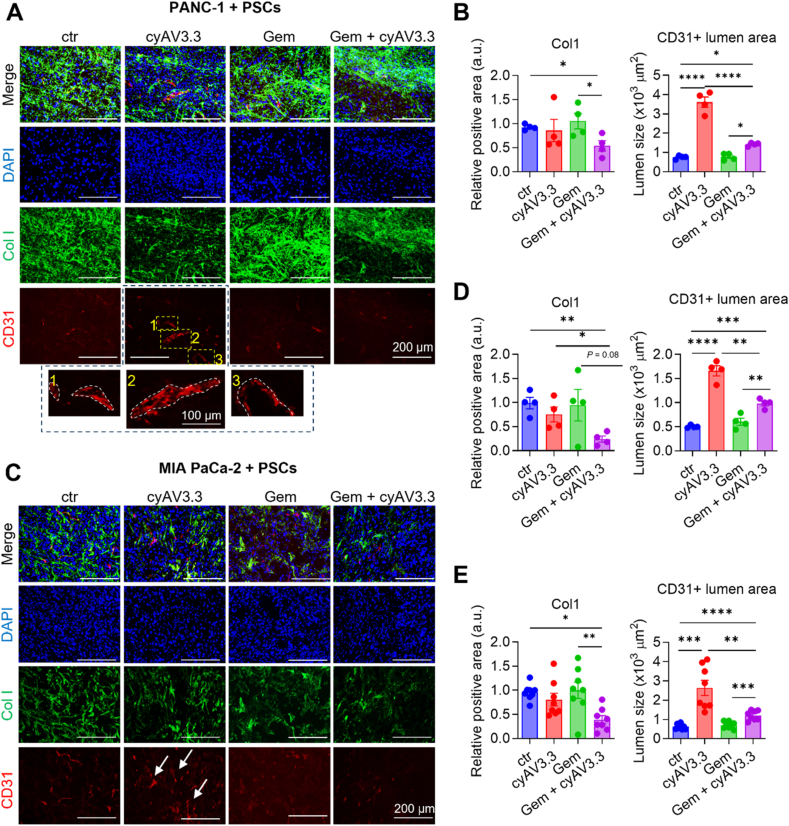
cyAV3.3 reduces collagen and decompresses blood vessels in gemcitabine-treated mice in co-injection xenograft models. (A) Representative immunohistochemistry for ECM protein collagen type I (green) and endothelium marker CD31 (red) in the PANC-1 + PSCs tumor model. Insets 1–3 show representative examples of the size of blood vessels. (B) Quantitation of collagen type I and CD31+ vessel lumen size in the PANC-1 + PSC tumor model. Data represent mean ± SEM, two-tailed unpaired Student's *t*-test. (C) Representative immunohistochemistry for ECM protein collagen type I (green) and endothelium marker CD31 (red) in the MIA PaCa-2 + PSCs tumor model. (D) Quantitation of collagen type I and CD31+ vessel lumen size in the MIA PaCa-2 + PSCs model. (E) Collective analyses of both tumor model. Data represent mean ± SEM, ordinary one-way ANOVA for multiple comparisons corrected with Holm-Sidak test. ∗*P* < 0.05, ∗∗*P* < 0.01, ∗∗∗*P* < 0.001, ∗∗∗∗*P* < 0.0001.

Furthermore, we analyzed the isolated tumors for the effects of cyAV3.3 on fibrosis (collagen type I deposition) and tumor vasculature (CD31 as an endothelial marker) using immunofluorescence staining. Immunofluorescence staining and quantitative data showed that the treatment with cyAV3.3 in combination with gemcitabine reduced the collagen expression in both tumor types (Fig. 6A–D). We also determined the collective effect in both tumor models, which resulted in a consistent outcome (Fig. 6E). Abundant tumor fibrosis results in the tumor vasculature compression in the PDAC, reducing the drug delivery^28^. We therefore examined the effect of the treatment on the blood vessel compression. The tumor blood vessel lumen diameter was measured digitally by drawing an overlay of the CD31+ lumen, as shown in Fig. 6A (see zoomed images). We found that blood vessels in the cyAV3.3-treated tumors showed larger lumen diameter compared to the control tumors (Fig. 6A–D). This decompression effect of cyAV3.3 was also found in the combination treated tumors, although it was lesser than cyAV3.3 treatment alone, which might be due to cytotoxicity induced by gemcitabine.

### 3.8 Effect of cyAV3.3 on chemotherapy efficacy in genetically engineered KPC model

**Figure 7:**
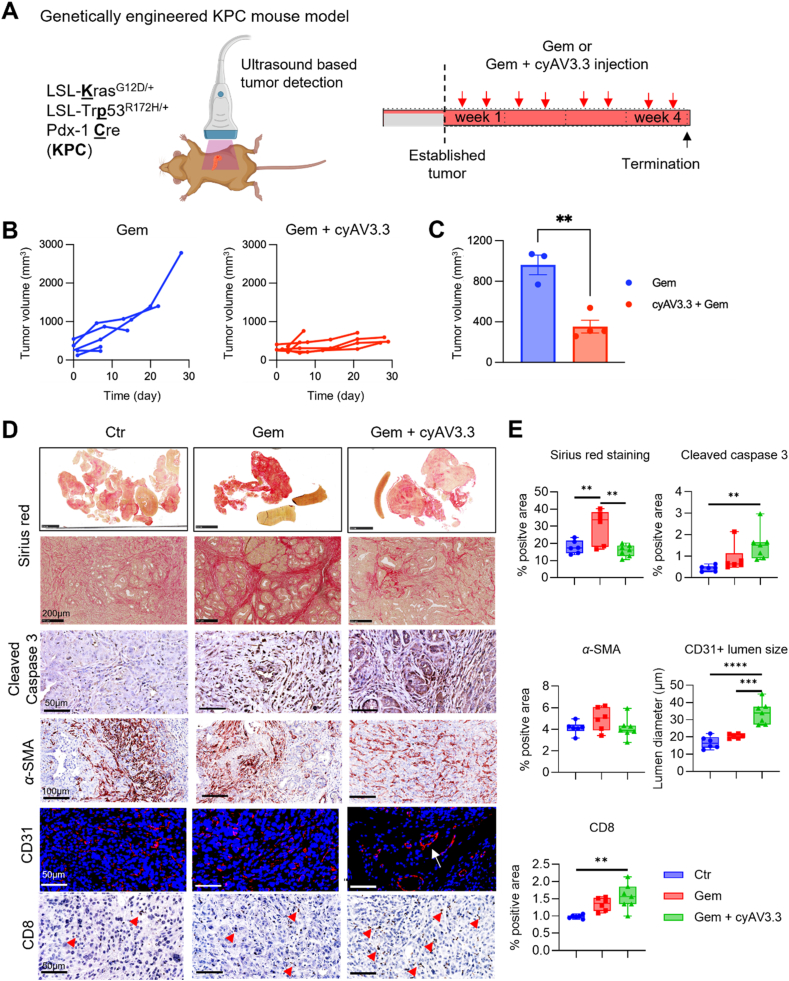
cyAV3.3 potentiates the effect of gemcitabine in the genetically engineered KPC mouse PDAC model. (A) Treatment regimen in the genetically engineered mouse model in *LSL-Kras*^G12D/+^; *LSL-Trp53*^R172H/+^; *Pdx1-Cre* mice. After tumor establishment (3–6 mm), determined by focused ultrasound measurement, animals were injected with either gemcitabine or combination of gemcitabine and cyAV3.3 twice a week for 4 weeks intraperitoneally. (B) Tumor growth of individual mouse at different timepoints treated either with gemcitabine or the combination of gemcitabine and cyAV3.3. The end of a line indicates the termination of mice due to extensive tumor growth or symptoms such as abdominal distension, loss of body conditioning, jaundice, hunching, or reduced movement. (C) Tumor size of animals at day 14 after tumor establishment. Mean ± SEM, ∗∗*P* < 0.01. (D) Representative immunohistochemistry and (E) quantification for extracellular collagen (Sirius red staining), cleaved caspase 3, *α*-SMA, cytotoxic T-cells (CD8, red arrow denotes positive cells), and the endothelial marker CD31 in the KPC mouse PDAC model. The bars represent mean ± SEM for 6 animals. Statistical analysis was performed as ordinary one-way ANOVA for multiple comparisons corrected with the Holm-Sidak test. ∗*P* < 0.05, ∗∗*P* < 0.01, ∗∗∗*P* < 0.001, ∗∗∗∗*P* < 0.0001.

After examining the effect of cyAV3.3 on the efficacy of gemcitabine in co-injection tumor models, we evaluated the efficacy of our cyclic peptide in the KPC spontaneous mouse model. After these tumors reached the size of 3–6 mm, mice were injected with gemcitabine alone or in combination with cyAV3.3 (Fig. 7A). Treatments were performed twice a week for up to 4 weeks and tumors were measured by ultrasound imaging every week. We found that cyAV3.3 in combination with gemcitabine reduced the tumor growth compared to gemcitabine alone (Fig. 7B and C).

Next, we examined the effect of treatment on collagen deposition in the isolated tumors using Sirius red staining. We found that gemcitabine significantly induced collagen deposition, as can be seen from the intense red color staining and bridge formation (Fig. 7D and E). Interestingly, combination with cyAV3.3 substantially reduced collagen deposition and fibrotic bridges. As seen in the co-injection tumor model, cotreatment with cyAV3.3 enlarged the tumor blood vessel lumen (Fig. 7D and E). We further investigated the level of apoptosis using cleaved caspase 3 staining, which showed an increase in apoptosis in the combination treated tumors (Fig. 7D and E). There was no change in the *α*-SMA + CAFs (Fig. 7D and E), but there was a reduction of their collagen producing activity.

## 4 Discussion

The tumor–stroma interaction drives the PDAC progression, promoting stemness and drug resistance in cancer cells, ultimately reducing chemotherapy efficacy. This study presents a novel cyclic peptide that block ITGA5-mediated interactions in CAFs, thereby inhibiting their own activation and ECM production, as demonstrated in human PSCs *in vitro*. In addition, this peptide disrupted CAF's pro-tumorigenic CAF–tumor cell interactions, reducing resistance and stemness markers, and enhancing efficacy of chemotherapy in stroma-rich 3D spheroid models. Furthermore, the radiolabeled cyclic peptide efficiently accumulated in tumors following different parenteral routes of administration *in vivo*. Notably, combining cyAV3.3 with gemcitabine significantly improved therapeutic efficacy in both co-injection xenograft models and a genetically engineered KPC mouse model of PDAC. These enhanced effects were associated with reduced desmoplasia, alleviated vasculature compression, increased CD8^+^ cytotoxic T cell infiltration, and enhanced apoptosis.

The role of CAFs in tumor progression, stemness and drug resistance is an interesting topic of research in recent years^29^^,^^30^. Depletion of *α*-SMA + CAFs using a conditional deletion leads to enhanced tumor progression in PDAC^31^^,^^32^. While these studies were based on genetic depletion rather than pharmacological inhibition of specific pathways in CAFs. The studies underline that rather than depletion of CAFs, their reprogramming from a pro-tumoral to an anti-tumoral phenotype is a preferable approach. In this study, we showed that cyAV3.3, instead of depletion, inhibited the differentiation and activation of CAFs, reducing the ECM production and crosstalk with cancer cells. Evidence also suggests that integrins interact with growth factors, and crosstalk between integrins and TGF-*β* signaling has been reported^14^^,^^33^, including regulation of *α*5*β*1 by TGF-*β*^34^ and vice versa^14^^,^^35^. In this study, blockade of *α*5*β*1 using cyAV3.3 led to the inhibition of TGF-*β*-induced pFAK in PSCs. These data are in line of previous research showing that FAK inhibition using small molecule inhibitors improved therapeutic efficacy of both chemotherapy and immunotherapy in different cancer types^36^^,^^37^.

Short linear peptides (less than 10 amino acids), such as AV3.3, are flexible in solution and thus less prone to interact with and bind to the pocket. Stabilizing the spatial structure of these biomolecules can be achieved by creating constrained structures such as *via* cyclization that limits the spatial degrees of freedom and improve their biological activity^38^^,^^39^. Our *in silico* molecular docking studies revealed that our peptides derived from FN docked at the same site as the native FN 9-10 units^40^. Furthermore, our blocking experiments confirmed that the peptides competed with *α*5*β*1 or PSCs in binding assays and showed the superiority of the cyclic version. Moreover, the inhibition of PSC differentiation and ECM production confirmed the higher potency of the cyclic peptide compared to the linear version.

In the native microenvironment, PSCs and CAFs are constantly activated by tumor cells *via* cell–cell contact and paracrine signaling^41^, as well as by ECM secreted by themselves, which turns the tumors stiff and promote stemness and chemoresistance^7^. As shown in our 3D spheroid models, heterospheroids of cancer cell lines with PSCs were more compact compared to their counterpart homospheroids and induced the genes related to resistance, stemness and ECM. Interestingly, treatment with cyAV3.3 significantly inhibited these genes in heterospheroids and potentiated the anti-tumoral effect of gemcitabine in heterospheroids. A recent study has shown that myofibroblasts in hepatocellular carcinoma can induce stemness and resistance by transferring ITGA5 to cancer cells^27^, which explains how our peptide may inhibit stemness and resistance genes in our studies. Except ABCG1 and KRAS, there was no effect on the gene expression in homospheroids, indicating that reduction in genes by cyAV3.3 was due to interruption of the tumor–stroma interaction. The inhibitory effect of cyAV3.3 on ABCG1 and KRAS in homospheroids might be due to the inhibitory effect of cell–cell interaction among tumor cells.

Our *in vivo* data confirmed that the radiolabeled cyAV3.3 rapidly accumulated into the tumor after either intravenous or intraperitoneal routes of administration in a stroma-rich PDAC mouse model. Of note, treatment with cyAV3.3 to the co-injection tumor cell models with different tumor cell lines, mimicking tumor heterogeneity, not only enhanced the efficacy of gemcitabine but also reduced the tumor growth as a monotherapy. The latter effect might be attributed to the inhibition of tumor–stroma interaction. Furthermore, the tumor growth arrest in the spontaneous KPC model confirmed the enhanced efficacy of gemcitabine in combination with cyAV3.3 due to reduced desmoplasia, increased apoptosis and enhanced CD8^+^ T cell infiltration. Of note, treatment with gemcitabine enhanced collagen expression: a pro-fibrotic effect of chemotherapy that has been shown previously^7^.

## 5 Conclusions

In conclusion, this study presents a novel cyclic peptide cyAV3.3 targeting the ITGA5 receptor, which was more effective than the linear peptides in inhibiting PSC differentiation. It also inhibited CAF-induced ECM deposition as well as CAF-cancer cell crosstalk mediated by ITGA5, responsible for chemoresistance and stemness. The ability of cyAV3.3 to enhance the efficacy of gemcitabine was successfully demonstrated in the 3D heterospheroid tumor models *in vitro*. Furthermore, both co-injection xenograft models as well as spontaneous KPC PDAC models robustly demonstrated the ability of the peptide to enhance the efficacy of chemotherapy. Overall, these findings support further investigation of cyAV3.3 as a potential adjuvant therapy to improve chemotherapy outcomes in pancreatic cancer and other malignancies with abundant stroma.

## Author contributions

Deby Fajar Mardhian performed, analyzed, and interpreted the experimental data and wrote the initial manuscript. Kunal P. Pednekar performed histological analysis of KPC model, while Praneeth Reddy Kuninty helped with animal experiments and other analysis. Ahmed G. Hemdan and Sabine de Winter contributed to different *in vitro* analyses. Saadia A. Karim performed genetic KPC tumor model. Josbert M. Metselaar contributed with PK study design. Jennifer P. Morton contributed with the study design and performance of the genetic KPC model as well as in editing the manuscript. Jai Prakash supervised the entire study including design of the study, interpretation of the results, writing and editing of the manuscript.

## Conflicts of interest

Jai Prakash is the founder and shareholder of ScarTec Therapeutics BV. Josbert M. Metselaar is a co-founder and shareholder in ScarTec Therapeutics. Jai Prakash is a co-owner and inventor of a patent filed by the University of Twente (WO2017069627A1). The other authors declare that they have no competing interests.

## Appendix A Supplementary data

The following is the Supporting Information to this article:
