# Supplementary material for: Engineered cyclic peptide targeting ITGA5 disrupts tumor–stroma interaction to overcome desmoplasia and resistance in pancreatic ductal adenocarcinoma
Source: Acta Pharm Sin B. 2025 Oct 24;16(1):305–21. doi: 10.1016/j.apsb.2025.10.022 (PMC12828148; doi:10.1016/j.apsb.2025.10.022)
Supplement: Multimedia component 1 [file mmc1.pdf]

Supporting Information for

Original article

Engineered cyclic peptide targeting ITGA5 disrupts tumor-stroma interaction to overcome desmoplasia and resistance in pancreatic ductal adenocarcinoma

Deby Fajar Mardhian[a,b], Kunal P. Pednekar[a], Ahmed G. Hemdan[a], Praneeth Reddy Kuninty[a], Saadia A. Karim[c], Sabine de Winter[a], Josbert M. Metselaar[d,e], Jennifer P. Morton[c,f], Jai Prakash[a,d,g,*]

[a]*Engineered Therapeutics Group, Department of Advanced Organ Bioengineering and Therapeutics, Faculty of Science and Technology, University of Twente, Enschede 7500AE, The Netherlands*

[b]*Department of Dental Materials and Technology, Faculty of Dentistry, Padjadjaran University, Bandung 40132, Indonesia*

[c]*CRUK Scotland Institute, Glasgow G61 1BD, UK*

[d]*ScarTec Therapeutics BV, Horst 2, Enschede 7522LW, The Netherlands*

[e]*Institute for Experimental Molecular Imaging (ExMI), RWTH Aachen University Hospital, Aachen 52074, Germany*

[f]*School of Cancer Sciences, University of Glasgow, Glasgow G61 1QH, UK*

[g]*Department of Medical Biosciences, Radboud University Medical Centre, Geert Grooteplein Zuid 10, Nijmegen 6525 GA, The Netherlands*

Received 25 February 2025; received in revised form 18 August 2025; accepted 26 August 2025

[*]Corresponding author.

E-mail address: jai.prakash@radboudumc.nl (Jai Prakash).

Running title: Cyclic peptide targeting CAFs in PDAC

Alanine replacement assay

Alanine was used as a replacement of each amino acid in the peptide sequence RYYRITY one by one using a peptide array (JPT GmbH). The array was exposed to the human recombinant $\alpha5\beta1$ receptor (RnD) as per the manufacturer's protocol, followed by incubations with mouse anti-human CD49e (RnD Systems). Then the array was washed and the

secondary peroxidase labeled antibody was added and incubated for 1 h at RT. The HRP signal was developed using standard chemiluminescence kit and the signal was measured.

Peptide stability

To evaluate the stability, 20 µmol/L peptide was incubated in 50% human plasma at biological temperature 37 ℃ for 1, 2, and 24 h. After incubation, plasma was precipitated by adding equal volume of 4% trichloroacetic acid (TCA) and rested on ice for 10 min. As control (or 0h), precipitation was performed immediately after mixing the peptide and plasma. Supernatant was collected after centrifugation at 13,300 × *g* and assessed using reversed phase high performance liquid chromatography (HPLC) with 5%, 95%, and 99.9% acetonitrile in de-gassed water with 0.1% trifluoroacetic acid as eluents. The remaining peptide was analyzed at a wavelength 280 nm.

Quantitative real-time PCR

To examine the effect of peptides on the gene expression of hPSC activation markers, cells were seeded into a 12 well plate at a seeding density of 50,000 cells/well. After overnight incubation, cells were starved for 24 h, then treated with human recombinant 5 ng/mL TGF-β (Peprotech, Hamburg, Germany) and 50 mmol/L peptide. After 24 h of the incubation, cells were lysed and total RNA was isolated using GenElute™ Mammalian Total RNA Miniprep Kit (Sigma–Aldrich) and the RNA concentration was measured using a NanoDrop® ND-1000 Spectrophotometer (Thermo Scientific, Waltham, USA). cDNA was synthesized with iScript™ cDNA Synthesis Kit (BioRad, Veenendaal, The Netherlands), and 10 ng cDNA was used for each PCR reaction. The real-time PCR primers (Table S1) were purchased from Sigma–Aldrich. Quantitative real time PCR was performed with 2× SensiMix SYBR and Fluoroscein Kit (Bioline GmbH, Luckenwalde, Germany) using a BioRad CFX384 Real-Time PCR detection system (BioRad). Gene expression levels were normalized to the expression of the house-keeping gene 18s rRNA.

Western blot analysis

To evaluate the effect of peptides on the protein expression of hPSC activation markers, hPSCs were seeded into a 12 well plate at seeding density 50,000 cells/well. The next day, the medium was changed with 0% FBS Stellate Cell medium to starve hPSCs for 24 h. Subsequently, hPSCs were incubated with 5 ng/mL TGF-β1 and peptide for 48 h to examine the effect on protein expression of different markers such as α-SMA and collagen-1. Cells were lysed using 1× blue loading buffer containing 1× DTT reducing agent (Cell Signaling Technology) and homogenized using ultrasonication. Protein lysates were separated on a 4%–20% Tris-Glycine gel (Thermo Scientific) and then transferred onto a PVDF membrane (Thermo Scientific). The blots were incubated with the primary antibody overnight at 4 ℃ followed by incubations with species specific HRP conjugated secondary antibody for 1 h at room temperature. Proteins were detected with

Pierce™ ECL Plus Western Blotting substrate kit (Thermo Scientific) and the membranes were exposed to FluorChem™ M System (ProteinSimple, San Jose, USA). The protein signals were quantified using ImageJ Software (NIH) and the target protein expression levels were normalized to β-actin.

Immunocytochemical staining

PSCs were seeded into 24 well plates at a seeding density of 10,000 cells/well. After overnight, cells were starved for 24 h and treated with 5 ng/mL TGF-β and each peptide at 20 µmol/L. After 48 h incubation, cells were fixed with acetone:methanol (1:1) for 30 min at –20 ℃ followed by drying at RT and rehydration with PBS. To analyze protein expression of α-SMA and collagen-1, cells were incubated overnight with respective primary antibody at room temperature. The next day, cells were washed with PBS and incubated with horseradish peroxidase (HRP)-labeled secondary antibody diluted in PBS supplemented with 5% vol of normal human serum. Next, cells were stained using AEC staining kit (Sigma–Aldrich) and cell nuclei were counterstained using hematoxylin. Finally, cells were washed with tap water and then mounted with AquateX mounting medium. Images were made using Nikon E300 microscope (Nikon). Images were analyzed using ImageJ software (NIH).

Immunohistochemical staining of mouse tumors

Tumors were cut into 7 µm thick sections and mounted on glass slides using Cryotome FSE (Thermo Scientific), dried, and fixed in acetone for 15 min. Tumor sections were rehydrated in PBS for 10 min then incubated with primary antibody diluted in PBS for 1 hour at RT. Antibodies details are given in Table S2. Next, slides were washed 3 times with PBS before incubation with secondary fluorescence-labeled antibody diluted in PBS. After 30 min of incubation, slides were washed with PBS and shielded with a DAPI-containing mounting medium. Images were made using a Hamamatsu NanoZoomer Digital slide scanner 2.0HT (Hamamatsu Photonics, New Jersey, USA).

Supporting Tables

Table S1 Predicted interaction between AV3.3 or cyAV3.3 and a5b1 receptor using molecular docking.

AV3.3			cyAV3.3		
AA	Residue ITGA5B1	Interaction	AA	Residue ITGA5B1	Interaction
Ac			G		
R	α5 I-225	Hydrogen bond	R		
	α5 I-225	Hydrogen bond			
	α5 D-227	Hydrogen bond			
	α5 S-229	Hydrogen bond			
	α5 S-229	Hydrogen bond			
Y			Y	β1 K-182	Hydrogen bond
				β1 P-186	Hydrogen bond
Y	β1 S-227	Hydrogen bond	Y		
	β1 F-262	Hydrogen bond			
R			R	β1 D-226	Hydrogen bond
A	β1 S-227	Hydrogen bond	A		
T	β1 S-134	Hydrogen bond	T	β1 S-227	Hydrogen bond
Y	α5 D-227	Hydrogen bond	Y	α5 D-227	Hydrogen bond
	β1 L-225	Hydrogen bond		α5 D-227	Hydrogen bond
			K		

Table S2 Details of the antibodies used in the study.

Antibody	Source	Dilution	
		IHC	IFC
Goat anti-type I collagen	Southern Biotech	1:300	1:300
Mouse monoclonal anti-actin, α-smooth muscle	Sigma–Aldrich	1:600	1:600
Rabbit monoclonal anti-phospho-FAK	Cell Signaling	1:1000	
Mouse monoclonal anti-FAK	Cell Signaling	1:1000	
Mouse monoclonal anti-β-actin	Sigma–Aldrich	1:5000	
Rat anti-mouse CD8a	Southern Biotech	1:100	
Rabbit polyclonal anti-cleaved caspase 3	Invitrogen	1:100	
Rat polyclonal anti-mouse CD31	Southern Biotech		1:50
Polyclonal rabbit anti-goat immunoglobulin HRP	Dako	1:2000	
Polyclonal goat anti-rabbit immunoglobulin HRP	Dako	1:2000	
Polyclonal goat anti-mouse immunoglobulin HRP	Dako	1:2000	
Polyclonal rabbit anti-mouse immunoglobulin HRP	Dako	1:2000	
Alexa Fluor® 488 donkey anti-goat IgG	Invitrogen		1:100
Alexa Fluor® 594 donkey anti-rat IgG	Invitrogen		1:100

Table S3 Sequences of forward and reverse primers used during real-time PCR.

Gene	Forward primer	Reverse primer
RP18	TGAGGTGGAACGTGTGATCA	CCTCTATGGGCCCGAATCTT
ACTA2	CCCCATCTATGAGGGCTATG	CAGTGGCCATCTCATTTTCA
COL1A1	GTACTGGATTGACCCCAACC	CGCCATACTCGAACTGGAAT
FN1	GTATACGAGGGCCAGCTCAT	CCCAGGAGACCACAAAGCTA
ITGA5	CAACTTCTCCTTGGACCCCC	GTCCTCTATCCGGCTCTTGC
ABCG1	TTCCCCTGAACATTCTCCCC	CATTCCTCACGGTGATGCTG
BCL2	GTCTGGGAATCGATCTGGAA	AATGCATAAGGCAACGATCC
CXCR4	GCGGTTACCATGGAGGGGAT	CCCATGACCAGGATGACCAAT
KRAS	GAGGCCTGCTGAAAATGACTG	ATTACTACTTGCTTCCTGTAGG
WNT1	CCTCCACGAACCTGCTTACA	TCCCCGGATTTTGGCGTATC
CD44	AGGAACCTGCAGAATGTGGA	GTTAAGTGTCCCAGCTCCCT
SHH	CAAGCTGGTGAAGGACCTGA	CGCGTCTCGATCACGTAGAA
MMP2	AGGAGGAGAAGGCTGTGTTC	CTCCAGTTAAAGGCGGCATC
MMP9	TCTTCCCTGGAGACCTGAGA	TTTCGACTCTCCACGCATCT
LOX	ATTTCTTACCCAGCCGACCA	ACTTGCTTTGTGGCCTTCAG

Supporting Figures

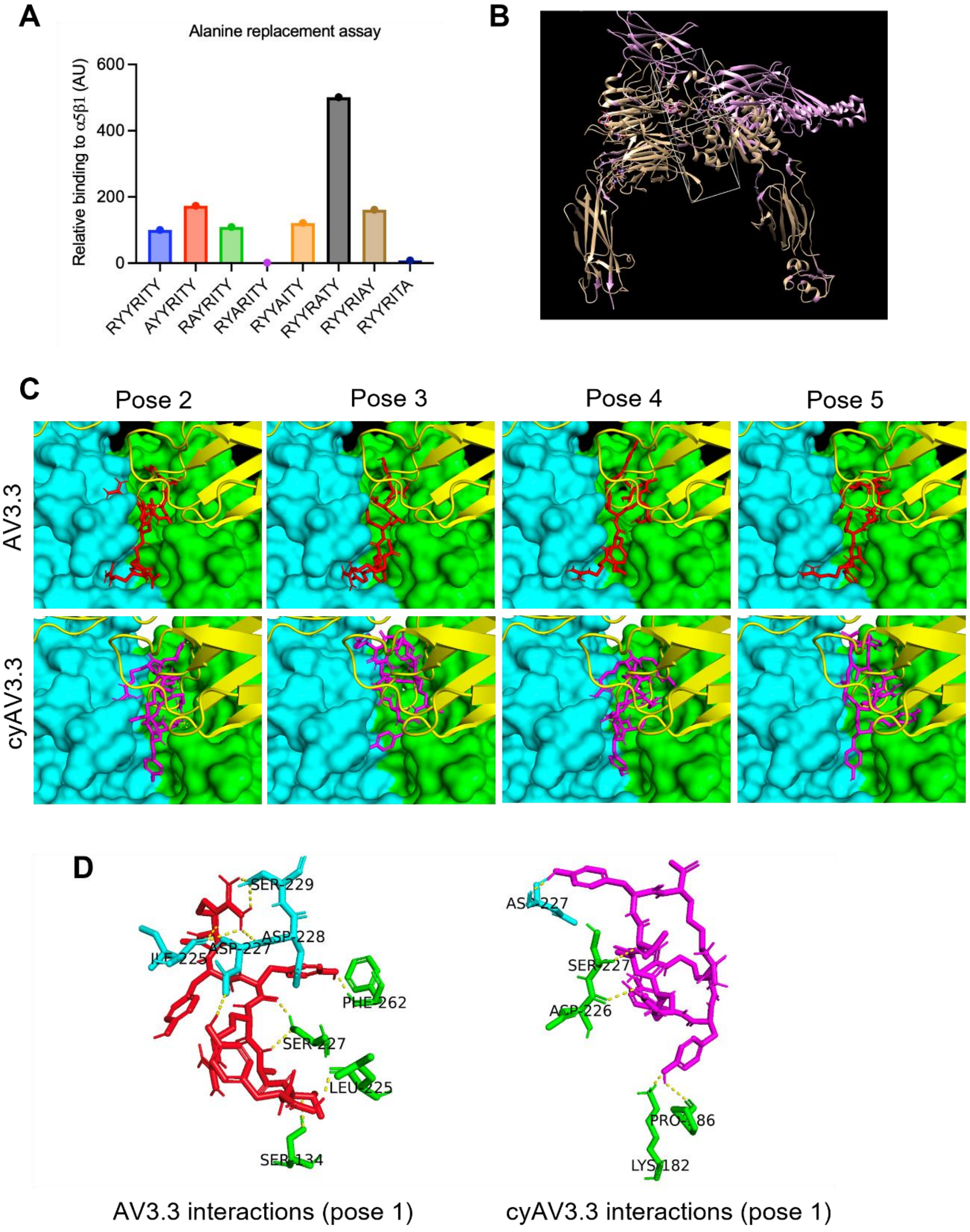

Figure S1 Predicted poses of AV3.3 and cyAV3.3 interaction with $\alpha5\beta1$ receptor using molecular docking. (A) Alanine replacement assay for the RYYRITY sequence for binding to recombinant $\alpha5\beta1$ receptor. (B) The grid was placed to cover the binding site at which

FN (domain 10) bound to the receptor in order to perform the docking analysis. (C) Docking structures showing the interaction poses of AV3.3 (red) and cyAV3.3 (magenta) with $\alpha5\beta1$. $\alpha5$ is depicted in blue while $\beta1$ in green color and FN in yellow color. (D) Structure showing interactions of amino acids from the peptides and interacting chains. The dotted lines show the polar contacts. The structure analysis was performed using PyMol software ver 2.5.5.

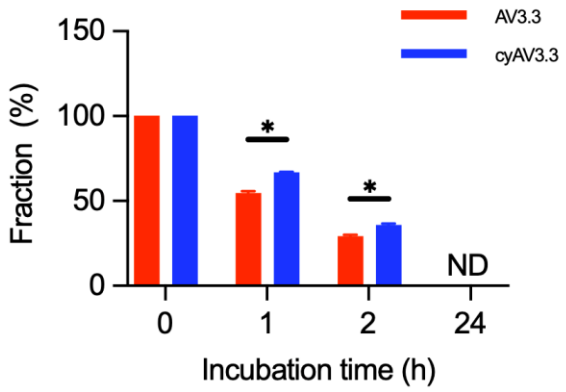

Figure S2 Stability analysis of AV3.3 and cyAV3.3 in human plasma after incubation at 1, 2, and 24 h.

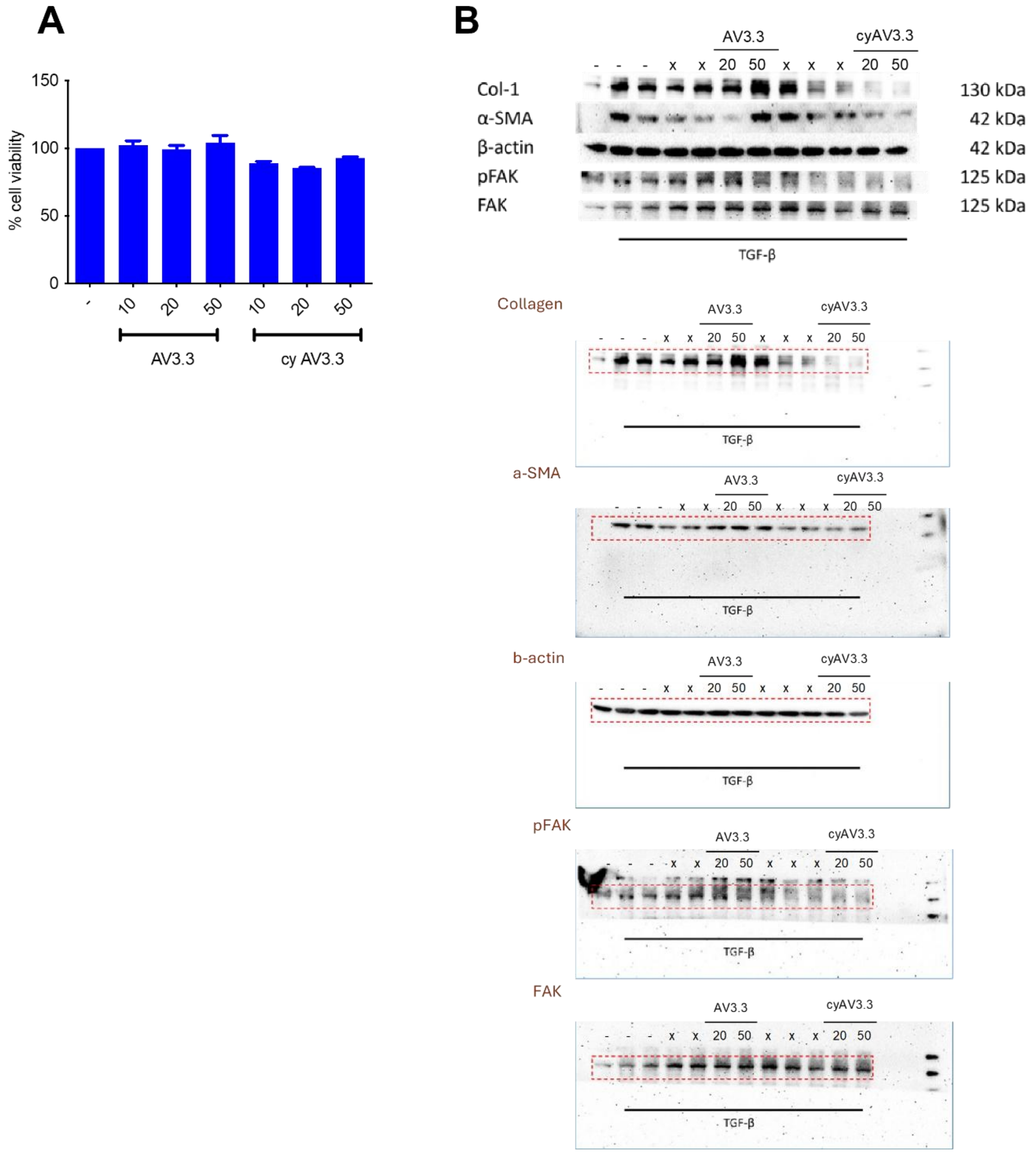

Figure S3 (A) Effect of AV3.3 and cyAV3.3 on the cell viability of hPSCs. (B) The original images of the Western blots for each protein. The symbol × represents lanes representing other samples which were run along.

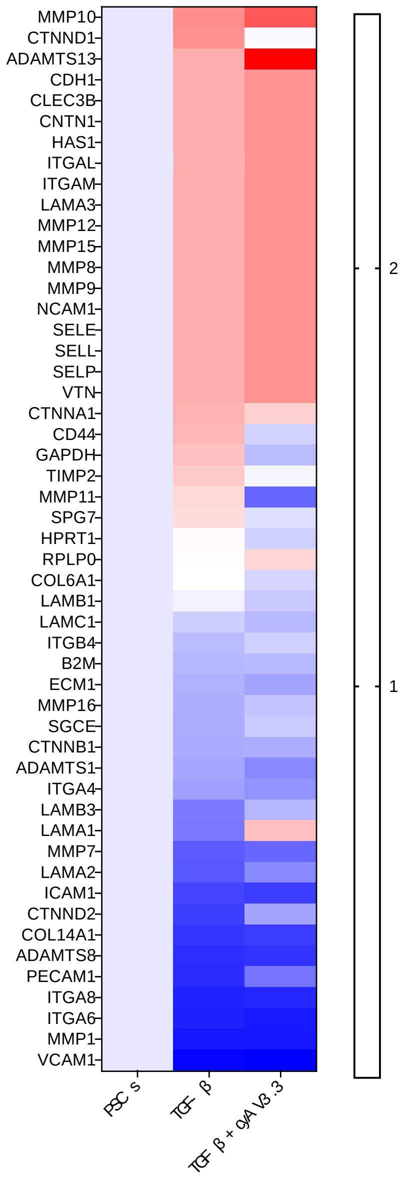

Figure S4 Heatmap showing the effect of cyAV3.3 on the TGF-*β* induced gene expression levels in hPSCs.

A

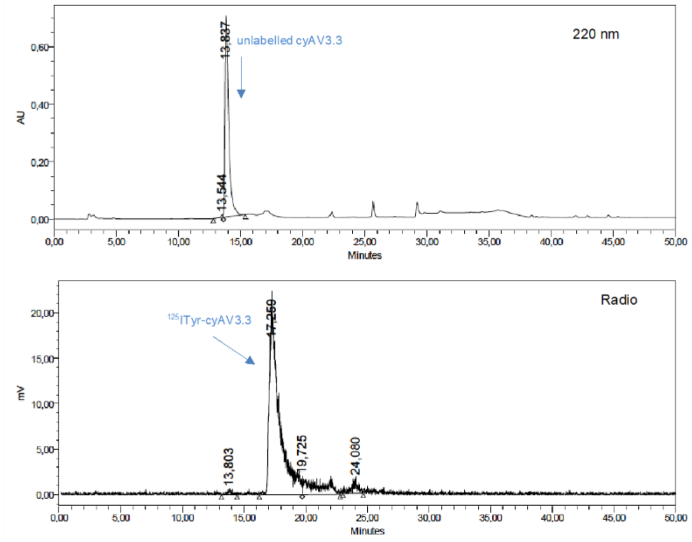

	RT (min)	Area (μV*sec)	% Area	Height (μV)
1	13,544	115073	0,76	10395
2	13,803	12865	0,93	676
3	13,837	14963365	99,24	701472
4	17,259	1157400	84,06	21153
5	19,725	160627	11,67	2030
6	24,080	46044	3,34	1806

B

- D3 analysis

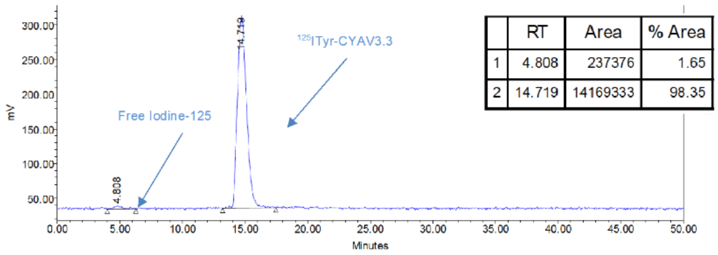

	RT	Area	% Area
1	4.808	237376	1.65
2	14.719	14169333	98.35

- D9 analysis

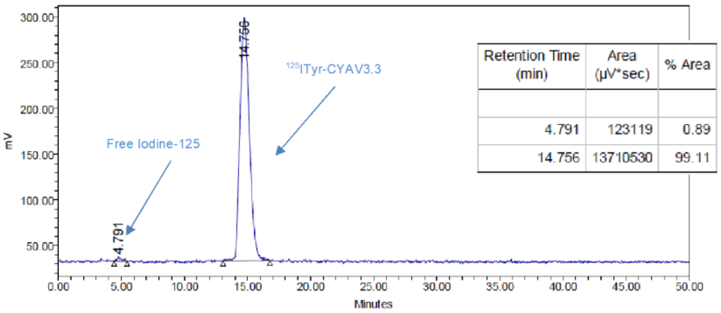

Retention Time (min)	Area (μV*sec)	% Area
4.791	123119	0.89
14.756	13710530	99.11

Figure S5 Chromatography analysis for radiolabeled cyAV3.3 characterization and purity. (A) The chromatograms showing the detection of peak for cyAV3.3 (upper graph) and a shift in the retention time after radiolabeling with iodine-125 as well as detection with gamma counter detector. The table shows that 99.2% was labeled. (B) HPLC chromatograms showing the peaks of the purified ^{125}I-cyAV3.3 with 98.4% pure product on Days 3 and 9 after storage in –20 °C.

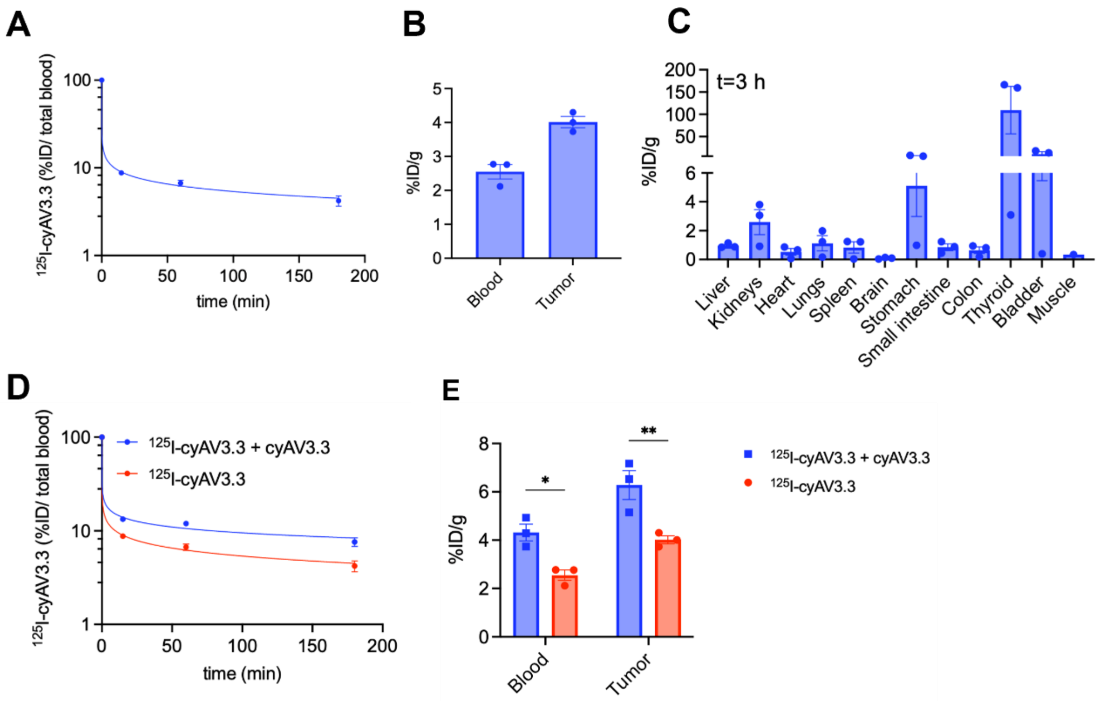

Figure S6 Biodistribution of radiolabeled cyAV3.3 and effect of unlabeled peptide. (A) PK profile of ^{125}I-cyAV3.3 after i.v. injection in PANC-1+PSC tumor-bearing xenograft mouse model. (B) Distribution in blood and tumor at 3 h after i.v. injection. (C) Percentage of injected dose per gram (%ID/g) (D, E) Effect of cold cyAV3.3 on the PK profile and tumor accumulation. Mice were treated with cold cyAV3.3 (4 mg/kg, i.v.) 10 min before the i.v. injection of ^{125}I-cyAV3.3. Data represent means ± SEM and the statistical analysis was performed using ordinary two-way ANOVA corrected for multiple comparison with Sidak test. **P* < 0.05. ***P* <0.01.

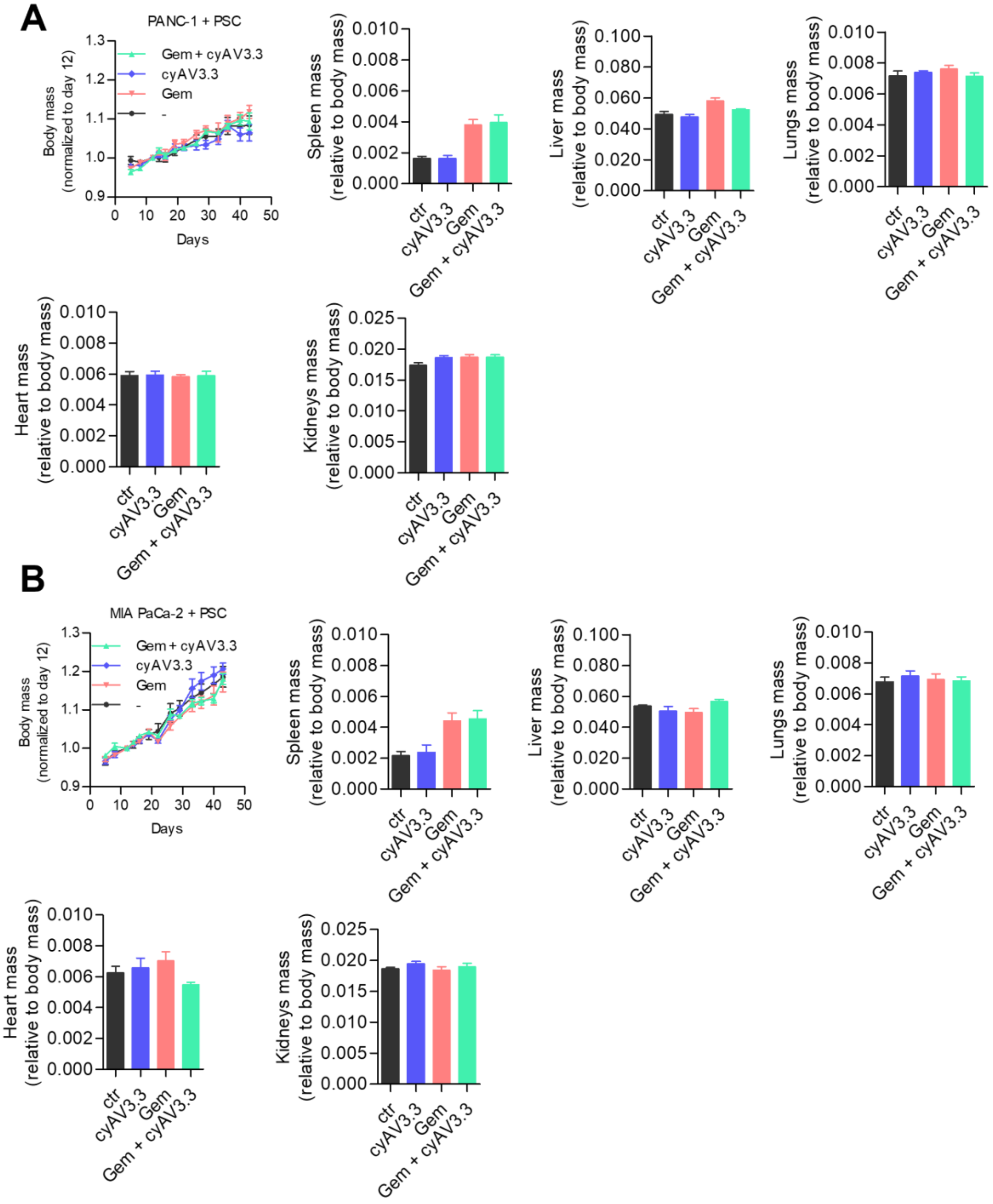

Figure S7 cyAV3.3 showed no side effects in co-injection tumor models. Body weight of mice during the animal experiment in the PANC1 + PSCs model (A) and the MIA PaCa-2 + PSCs model (B). All body weight is normalized to animal's body weight on Day 12. No loss of body weight was observed. Panels show weight of spleen, liver, lungs, heart, and kidneys. All organ weight is normalized to body weight. No side effect caused by cyAV.3

was observed according to the organ weight and body weight. The bars represent mean ± SEM.
